# Covalent Strategies for Targeting Messenger and Non-Coding RNAs: An Updated Review on siRNA, miRNA and antimiR Conjugates

**DOI:** 10.3390/genes9020074

**Published:** 2018-02-06

**Authors:** Santiago Grijalvo, Adele Alagia, Andreia F. Jorge, Ramon Eritja

**Affiliations:** 1 Institute of Advanced Chemistry of Catalonia (IQAC, CSIC), Jordi Girona 18-26, 08034 Barcelona, Spain; 2 Networking Centre in Bioengineering, Biomaterials and Nanomedicine (CIBER-BBN), Jordi Girona 18-26, 08034 Barcelona, Spain; adele.alagia@gmail.com; 3 Coimbra Chemistry Centre, (CQC), Department of Chemistry, University of Coimbra, Rua Larga, 3004-535 Coimbra, Portugal; andreiaj@qui.uc.pt

**Keywords:** covalent approach, gene delivery, gene therapy, non-coding RNA, miRNA technology, nucleic acid conjugates, RNA interference

## Abstract

Oligonucleotide-based therapy has become an alternative to classical approaches in the search of novel therapeutics involving gene-related diseases. Several mechanisms have been described in which demonstrate the pivotal role of oligonucleotide for modulating gene expression. Antisense oligonucleotides (ASOs) and more recently siRNAs and miRNAs have made important contributions either in reducing aberrant protein levels by sequence-specific targeting messenger RNAs (mRNAs) or restoring the anomalous levels of non-coding RNAs (ncRNAs) that are involved in a good number of diseases including cancer. In addition to formulation approaches which have contributed to accelerate the presence of ASOs, siRNAs and miRNAs in clinical trials; the covalent linkage between non-viral vectors and nucleic acids has also added value and opened new perspectives to the development of promising nucleic acid-based therapeutics. This review article is mainly focused on the strategies carried out for covalently modifying siRNA and miRNA molecules. Examples involving cell-penetrating peptides (CPPs), carbohydrates, polymers, lipids and aptamers are discussed for the synthesis of siRNA conjugates whereas in the case of miRNA-based drugs, this review article makes special emphasis in using antagomiRs, locked nucleic acids (LNAs), peptide nucleic acids (PNAs) as well as nanoparticles. The biomedical applications of siRNA and miRNA conjugates are also discussed.

## 1. Introduction

Classical therapeutic approaches and conventional drug discovery programs have been aimed at designing ligands and small molecule drugs capable of interacting and inactivating the function of a particular protein. In addition to traditional drugs [1], many efforts have been carried out in the search for novel pathways in which non-canonical drugs could be an alternative therapy [2]. The concept of antisense therapy was developed by Zamecnik and Stephenson in the late of 70 s when they showed the ability of an antisense oligonucleotide (ASO) to inhibit the RNA translation of the Rous sarcoma virus [3]. The ability of the oligonucleotides to electrostatically interact with target mRNAs and therefore block the translation process of a given protein has yielded new perspectives in the treatment of gene-related diseases.

In addition to ASOs, significant progress has been made in the field of RNAs that has led to the emergence of other entities capable of interacting specifically with mRNAs (e.g., siRNAs) as well as targeting other kinds of RNA molecules which are not translated into proteins (non-coding RNAs; ncRNAs) (e.g., miRNAs). Both strategies enabled the modulation of the RNA function by modifying gene expression levels and consequently the identification of novel DNA and RNA entities as promising therapeutic platforms and potential targets for drug development [4,5]. The formation of DNA:RNA and RNA:RNA hybrid complexes obtained from annealing complementary oligonucleotide sequences with target mRNA is the easiest way a priori to regulate gene expression and therefore inhibit the synthesis of proteins. Despite the existence of extensive knowledge of action mechanisms, these strategies sometimes exhibit lower silencing activities than expected. This loss of therapeutic efficacy is mainly caused by low stability in oligonucleotides in the bloodstream, innate immune system stimulation and its inability to impart cellular uptake among other side effects. These important hurdles have remarkably reduced the possibility of developing nucleic acids as promising therapeutic drugs and as an alternative to classical approaches [6]. Some of these deficiencies have been overcome by increasing the nuclease stability of nucleic acids and immunogenicity by chemically modifying nucleobases, sugar as well as their internucleotide linkage, respectively [7]. Despite advances in improving not only nuclease stability but also biodistribution and pharmacokinetic properties of nucleic acids without losing efficacy, the efficient administration of oligonucleotides for targeting cells is clearly essential to making the gene silencing process function properly. To overcome this barrier, both formulation and covalent approaches have emerged as promising alternatives for promoting gene delivery in contrast with the use of viral vectors [8,9]. Thus, defined non-viral vectors like cell-penetrating peptides (CPPs), polymers and liposomes (among others) have been electrostatically combined or covalently conjugated with nucleic acids to improve both cellular uptake in the nucleus or cytoplasm. In addition, it would increase their biological half-life in serum improving its in vivo pharmacokinetic properties.

This extensive research focuses on developing strategies to obtain more stable oligonucleotides along with functionalizing materials with the potential to facilitate gene delivery. It has resulted in the launching of numerous nucleic acid-based therapeutic candidates into the clinic [4,10,11,12,13]. Since nucleic acid-based therapeutics were implemented in clinical studies some time ago, six nucleic acid drugs (Vitravene, Macugen, Mipomersen, Eteplirsen, Defibrotide and Nusinersen) have been approved by the FDA [14]. Numerous ASOs and siRNAs are found at Phase III whereas the rest of siRNA and miRNA candidates are, in most cases, at Phase I and II clinical trials. These studies cover a range of diseases including wet age-related macular degeneration just to name a few. Some representative oligonucleotide-based therapeutics are listed on Table 1.

**Figure 1:**
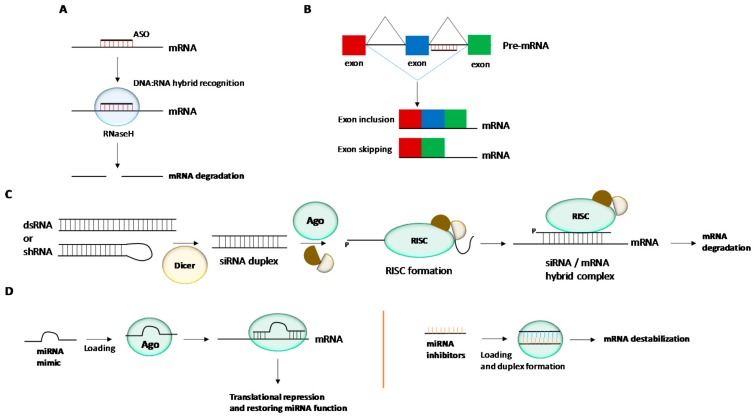
Gene regulation mechanisms mediated by antisense oligonucleotides (ASOs), triplex-forming oligonucleotides (TFOs), small interference RNAs (siRNAs) and microRNAs (miRNAs). (**A**) Antisense technology: RNAseH is able to recognize DNA:RNA hybrid complexes which leads to mRNA degradation and the blockade of protein synthesis. (**B**) RNA splicing mechanisms in order to restore the disrupted reading fragment of a gene by using ASOs targeting intron and exon junctions. (**C**) RNA interference: The siRNA duplex is unwound and the guide strand is recognized by Ago2. The resultant RISC complex is able to interact with complementary sequence of a target mRNA. (**D**) MiRNA technology: Gene expression can be modulated by using double or single-stranded miRNA mimics in order to restore the miRNA function by inhibiting translation process. MiRNA inhibitors with complementary sequences to miRNAs can also be used for inactivating ncRNAs.

Several mechanisms used by cells have been extensively studied which deal with the increase or decrease of protein production. This process is termed gene regulation and can be triggered by different pathways:

**Antisense technology.** This therapeutic strategy is based on hybridizing target mRNAs with oligonucleotides through Watson-Crick base-pair interactions [15,16]. ASOs are usually fully modified with phosphorothioate bonds (PS). The presence of such PS moieties throughout the entire oligonucleotide sequence confers some level of stability against endo and exonucleases. In this way, once a regular ASO is bound to a complementary target mRNA, RNaseH activation is initiated giving rise to mRNA degradation and the consequent inhibition of a given protein production (Figure 1A). The antisense activity can be also produced without activating the endonuclease enzyme cleavage via non-degrading mechanisms. The correct design of ASOs enables the steric ribosomal blockage when binding to the 5′-termini of targeting RNA sequences close to the intron-exon junctions giving rise to exon inclusion or exon skipping processes (Figure 1B). This efficiently blocks the translation processes and thus obtains the expected therapeutic effect.

**RNA interference (RNAi).** Fire and Mello’s discovery in the nematode *Caenorhabditis elegans* in which double-stranded RNAs (dsRNAs) can trigger the silencing of up-regulated genes was reported in 1998 and was finally awarded the Nobel Prize in Physiology or Medicine in 2006 [17]. The RNAi pathway is initiated by the ribonuclease Dicer protein which binds to and breaks dsRNAs in plants or short RNAi hairpin RNAs (shRNAs) in mammalian cells into small double-stranded fragments of (siRNAs). This siRNA duplex is primarily associated with the RNA-induced silencing complex (RISC). Then the duplex is unwound and the passenger (sense) strand is nicked by the Argonaute 2 (Ago2) protein. Additionally, the guide (antisense) strand is loaded into the RISC complex by the RISC-loading complex (RLC). The active RISC complex containing the guide strand is used to trigger post-transcriptional gene silencing through Watson-Crick base pair interactions with the target mRNA sequence. In particular, the 5′-termini of the siRNA guide strand is bound to the PIWI domain of Ago 2 whereas the 3′-termini is recognized by the PAZ domain. This causes a break of the targeted mRNA at positions 10 and 11 of the siRNA guide strand leading to the degradation of mRNA by cellular exonucleases and consequently stops the translation process (Figure 1C).

After confirming synthetic siRNAs can also trigger the RNAi process in mammalian cells [18], the importance of this regulation mechanism has greatly impacted basic and applied research, making the RNAi process one of the most promising tools for the treatment of human diseases. Despite the fact that RNAi has remarkably proved to be effective in vitro, important hurdles still need to be overcome in vivo when targeting aberrant proteins. In this regard, in addition to the problems associated with improving gene delivery and subsequent intracellular trafficking processes, there are other issues like unwanted OFF-target and immunogenicity effects which have deprived siRNA-based therapy from being an effective alternative to classical therapeutic approaches [19].

**miRNA technology.** A miRNA molecule is a small ncRNA made up of an oligonucleotide track containing about 21–23 nucleotides and is expressed in plants and animals. MiRNAs are involved in a good number of physiological processes like RNA silencing and post-transcriptional regulation of gene expression after binding at the 3′-untranslated region (3′-UTR) of specific mRNAs [20]. NcRNAs have demonstrated they play a role in human diseases and are considered to be therapeutic targets for the development of novel RNA-based therapies [21,22]. There are several ways to inhibit gene expression by targeting miRNAs [23] (Figure 1D): (i) The use of single-stranded oligonucleotides complementary to a miRNA sequence (antagomirs or anti-miRs); (ii) The use of double-stranded microRNA mimics; (iii) The use of small molecules that can act as miRNA inhibitors and (iv) The use of miRNA sponges.

In general, antisense technology has shown to be one of the most employed strategies for targeting miRNAs. This is produced by designing oligonucleotides (anti-miRs) fully complementary to miRNA leading to the formation of ASO:RNA duplexes with high stability. The use of miRNA mimics is similar to the siRNA molecule. The pre-formed duplex is loaded into RISC and causes the inhibition of the endogenous target mRNA after transfection. To improve this delivery, anti-miRs and miRNA mimics have been chemically modified in order to improve stability and biodistribution properties and therefore enhance their therapeutic applications [24,25]. Such proposed modifications will be reviewed below. The design and introduction of chemical modifications have been proposed and successfully evaluated for RNAi therapy and antisense technology (e.g., 2′-fluoro, 2′-methyl and 2′-methoxyethyl derivatives) as well as other promising anti-miRNA antisense constructs (e.g., ASOs, locked nucleic acids (LNAs) among others). Small molecules can be also used as miRNA inhibitors which have been mainly obtained from high-throughput screening (HTS) platforms and others [26,27]. These libraries of miRNA modulators interfere with the miRNA machinery at three different steps (e.g., primary miRNA (pri-miRNA) transcription, inhibiting Dicer or Drosha cleavage steps and impeding target mRNA recognition). The efficient blockage at each step might inhibit or restore the miRNA function involved in a variety of diseases suggesting the therapeutic potential for targeting miRNAs [28]. The third method reported to promote a miRNA loss-of-function is based on using miRNA sponges [29]. This approach is intended for the design of miRNA constructs containing specific binding sites (between four and ten positions) for certain miRNA seed regions which in turn, are able to block same families of ncRNAs. This particularity can offer interesting advantages in terms of selectivity for more miRNAs when compared to the design of anti-miR conjugates which are only aimed at targeting a single ncRNA.

Taking into account the importance of the gene expression regulation and its potential in the search for novel therapeutic agents, this article has placed special emphasis on the design of covalent strategies for delivering siRNA and miRNA conjugates reported in the last seven years. In the case of siRNA delivery, this review has been divided according to the non-viral vector used (e.g., CPPs, carbohydrates, polymers, lipids and aptamer chimeras). For a more detailed view on the field of chemical modifications and the strategies carried out for launching siRNAs into the clinic, several articles have been recently published [9,30,31]. In the second part of this article, we aimed to describe some relevant strategies based on modulating gene expression by delivering chemically modified anti-miRNAs (e.g., antisense oligonucleotides, locked nucleic acids (LNAs), peptide nucleic acids (PNAs) and other kinds of nanoconstructs). In particular, we have placed particular emphasis on using anti-miRNAs for targeting oncogenic ncRNAs. Detailed revisions based on targeting tumour-suppressive miRNAs can be also found in excellent reviews reported by Gambari [32] and Asghari [33].

## 2. Chemical Modifications and siRNA Delivery

**Cell-penetrating peptides—siRNA conjugates.** Cell-penetrating peptides (CPPs) are a family of short cationic peptides that have been employed as delivery vehicles for a good number of small molecules [34] and macromolecules (e.g., antibodies, proteins or nucleic acids) [35]. CPPs may come from natural or synthetic sources [36] and have the ability to cross biological barriers like plasma membrane and blood-brain barrier, among others. This capability displayed by the majority of CPPs is mainly determined by electrostatic interactions between guanidinium groups and sulphate, phosphate or carboxylate groups located in the cell membrane. These electrostatic interactions are the foundations for the formation of ion-pair complexes which may lead to cellular internalization of the complex by diffusion through the membrane. Formulation has become a widely used approach for the electrostatic complexation of nucleic acids with CPPs, especially with siRNAs. This strategy was used for the first time in the late 1990s [37] and continues to be one of the most promising alternatives for improving nucleic acid delivery [38]. However, the low stability of such siRNA polyplexes in extracellular and intracellular environments may cause a loss of efficiency in siRNA delivery. Extensive studies have been carried out in order to overcome this issue making emphasis on how the binding properties between CPPs and siRNAs could be improved.

A detailed study recently reported by Pärnaste et al. involving Transportan10 and a series of CPPs containing hydrophobic residues (e.g., PepFect and NickFect) [39] were complexed with a model siRNA sequence. The parameters that involve the CPP/siRNA complex formation as well as stability to degradation were thoroughly studied by different techniques like gel electrophoresis, isothernal titration calorimetry (ITC) and degradation experiments among others. As a result, the success of achieving optimal siRNA delivery was directly related to the pH changes effect caused on the overall positive charge of the resultant complex. Moreover, the presence of amphipathic tails in the CPP structure allowed siRNA complexes to display superior stability when compared to unmodified CPPs [40].

Covalent strategies have also been used for conjugating siRNA oligonucleotides with CPPs to overcome the polyplex dissociation risk in the bloodstream. This strategy helped obtain novel CPP-siRNA conjugates with promising silencing activities both in vitro and in vivo in the absence of transfection agents. Consequently, Tat [41], Penetratin [42], Transportan [43] and the lytic melittin peptide [44] have been selected as natural and artificial peptides for efficient siRNA delivery [45]. Other synthetic CPPs have been successfully conjugated to siRNA molecules. Recently, Yang et al. designed a strategy to conjugate low molecular weight protamine (LMWP: VSRRRRRRGGRRRR) with siRNA oligonucleotides [46]. The authors decided to use a bifunctional PEG spacer which connected both to the cationic peptide and the siRNA through a disulphide linkage. Ideally, once both siRNA conjugates were transfected and internalized, the resultant S-S bond is able to hydrolyse because of the presence of high levels of glutathione in the cytosol which results in the detachment of the peptide from siRNA [47]. To confirm this, the same authors also synthesized and characterized other kinds of CPP-siRNA conjugates containing a non-hydrolysable maleimide-sulphide linkage for comparison purposes. As expected, the siRNA potency exhibited by the two conjugates differed by virtue of being modified by either reducible or non-cleavable linkers, being the reducible one more effective. The disparity in the RNAi activity was not observed in cell internalization experiments which demonstrated similar efficiencies according to their confocal images and fluorescence intensities. Interestingly, the authors also confirmed the covalent strategy was more efficient in terms of cellular uptake than formulation approaches obtaining 2.3-fold increase when compared to the internalization efficiencies mediated by the corresponding lipoplex made of commercially available cationic lipids and siRNA.

GV1001 is a peptide which corresponds to residues 611–626 (EARPALLTSRLRFIPK) of the human telomerase reverse transcriptase (hTERT) protein. Initially, this peptide was used for stimulating an immune response against several cancers in an injectable cancer vaccine and is currently found under clinical studies [48,49,50]. In addition to exhibiting potent anti-cancer activities as a drug, other important biological activities have been reported (e.g., antioxidant [51], anti-inflammatory [52,53] and antiviral [54]). Recently, the use of GV1001 have been reported as protection of stem cells by mimicking hTERT protein [55] Lee et al. studied and characterized the cell entry mechanism of the peptide GV1001 [56]. The authors found that the peptide was able to mediate cellular uptake through endocytosis mechanisms via Heat Shock Protein (HSP) 70 and HSP90, which are primarily overexpressed and localized in the plasma membrane of tumour cells. This was confirmed when anti-HSP70 and anti-HSP90 antibodies were employed as competitive ligands leading to a decrease in peptide internalization. These results led them to use the GV1001 peptide as a CPP in order to deliver macromolecule therapeutics (e.g., proteins, DNA and siRNA). Specifically, the authors carried out covalent conjugation of GV1001 in a siRNA targeting luciferase gene. The effectiveness of this conjugation was compared by formulating the same siRNA with poly-lysine (PLL; 15-mer). In all cases, the GV1001-siRNA conjugate was able to silence luciferase more efficiently than their siRNA poliplex counterpart indicating the feasibility of these kinds of conjugates for anti-cancer drug delivery approaches.

The combination of liposomes with siRNA conjugates has been also reported to increase both stability and delivery efficacy of such macromolecules. Fan et al. were able to conjugate the peptide KALLAL into two siRNAs that were targeted against MEK1 mRNA (siMek1) and mutant B-RAF mRNA (siMB3), respectively [57]. In particular, KALLAL peptide was introduced at the 3′-termini of both passenger and guide strands according to solid-phase approaches. KALLAL-siRNA conjugates were able to effectively silence the corresponding protein expression for long periods of time in vitro and displayed excellent serum stabilities (up to 80%) that increased up to 21-fold when compared to naked siRNA. Additionally, KALLAL-siRNA conjugate showed greater effectiveness which was reflected in increasing the endosomal release effect when compared to oligofectamine. Promising results were also obtained in vivo. The oligonucleotide-peptide conjugate was able to significantly inhibit xenograft tumour growth in athymic mice, prolong circulation time and reduce its accumulation in kidneys. These results have shown the potential of this kind of modification in cancer therapy.

Related to this approach, Liu et al. have carried out extensive research for improving the gene delivery capacities of CPP-siRNA conjugates by combining thermal and magnetic dual-responsive liposomes (TMLs) with magnetic fluid Fe_3_O_4_ [58]. Additionally, the authors efficiently designed thermo-responsive liposomes (TSLs) containing NGR (AsnGlyArg) peptide and folate-targeted acid sensitive polymeric micelles (F-ASPM) as targeting moieties [59] in order to enhance target drug delivery by destabilizing TSLs in the acid tumour zone [60]. These strategies also allowed the authors to increase the stability of CPP-siRNA conjugates in vivo. In all cases, the authors chose a CPP derived from Penetratin (CKRRMKWKK) in order to assure membrane translocation efficiency whereas the selected siRNA was designed to downregulate c-*myc* oncogene, which is overexpressed in several cancer tumours. The siRNA was easily conjugated into the CPP through disulphide bonds to facilitate the dissociation of the corresponding siRNA in the cytosol. The authors reported in vitro gene silencing efficiencies as well as in vivo targeted delivery experiments in HT-1080 xenograft murine models mediated by CPP-siRNA conjugates when formulated into TML, TSL [59] and polymeric micelles [60], respectively. Results showed that external stimuli like magnetic fields and hyperthermia combined with the presence of folate receptors were able to prolong the efficacy of the conjugate and therefore reduce c-*myc* expression levels. Furthermore, this strategy allowed them to retard the tumour progression in vivo. These therapeutic strategies could open up new prospects in the synthesis of novel siRNA materials involving the covalent combination of CPPs.

**Carbohydrate—siRNA conjugates.** The covalent combination between carbohydrates and siRNA oligonucleotides has afforded promising nucleic acid conjugates with excellent properties not only in stability but also in promoting gene delivery. Carbohydrates participate in a good number of biological processes such as signal trafficking, cell surface recognition through lectins and carbohydrate-binding proteins [61,62]. The synthesis of carbohydrate-oligonucleotide conjugates has been thoroughly reported in literature [63,64]. Galactose (Gal) has become the most popular carbohydrate used for increasing the delivery of siRNA oligonucleotides especially for hepatocyte targeting delivery. For example, Ikeda et al. successfully developed a simple synthetic strategy based on the use of a cyclopentyl derivative, as a building block which was covalently linked at the carbohydrate anomeric position. Following this strategy, the authors were able to prepare siRNA conjugates containing one single Gal residue either at the guide (antisense) or passenger (sense) strand and also one additional conjugate with its both 3′-strands modified with the Gal residue [65]. The presence of cyclopentyl modification at the 3′-ends clearly stabilized the siRNA conjugates (up to 24 h) in 10% serum buffer at 37 °C when was compared with an unmodified siRNA. All carbohydrate-siRNA conjugates prepared by the authors were able to silence Surviving mRNA at 10 and 50 nM, respectively. The authors showed distinct gene silencing behaviour when either the passenger or guide strands were modified with Gal targeting group at the 3′-termini. While the modification of the passenger strand displayed comparable silencing activities to the unmodified siRNA, the modification of both strands slightly reduced the silencing activity obtaining similar efficacy than that observed for the siRNAs with the guide strand modified at the 3′-termini (up to ~80% of inhibition).

In addition to the 3′-termini, Gal derivatives were introduced at the 5′-termini using phosphoramidite approaches. A small series of carbohydrate derivatives fully modified with OMe moieties were incorporated at the 5′-termini of the siRNA passenger strand. This strategy reported by Vengut-Climent et al. [66] gave rise to the formation of three siRNA conjugates containing either one or two Gal derivatives. All conjugates were designed for targeting *Renilla* luciferase. Such modifications were recognized by the RNAi machinery when they were formulated with lipofectamine. Interestingly, these carbohydrate conjugates exhibited resistance to 5′-exonuclease up to 72 h after incubation with bovine spleen phosphodiesterase (BSP). The authors also evaluated the capacity of the conjugates to impart cellular uptake without using lipofectamine. These experiments only revealed that the double-tailed permethylated carbohydrate siRNA conjugate was able to moderately inhibit gene expression up to ~20%.

**Figure 2:**
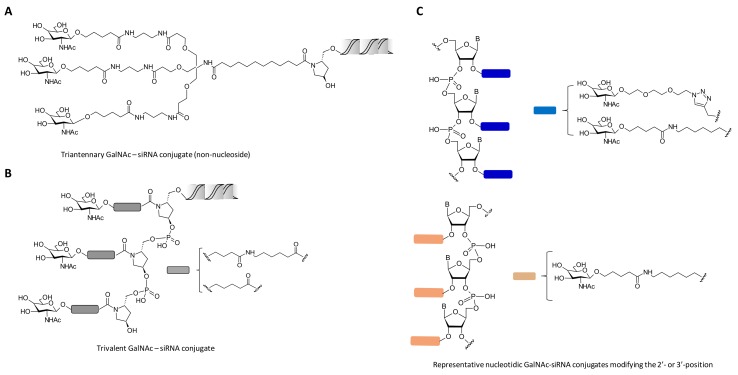
Schemes of carbohydrate_siRNA conjugates containing one triantennary GalNAc (**A**), three consecutive GalNAc residues (**B**) and nucleotides carrying monovalent GalNAc units at different positions of the siRNA duplex (**C**). Modifications were introduced at the 3′-termini of the siRNA passenger strand.

Other kind of carbohydrate architectures based on one, two and four Gal residues have been proposed by Aviñó et al. with the aim of modifying the 5′-termini of the siRNA passenger strand. The authors carried out the synthesis of a small series of carbohydrate-siRNA conjugates which were designed for inhibiting tumour necrosis factor-α (TNF-α) production and also for enhancing the cellular internalization of siRNAs [67]. TNF-α plays an important role in many inflammatory diseases and has been proposed to be a therapeutic target for Crohn’s disease, diabetes and obesity among other diseases. The authors showed that all carbohydrate-siRNA conjugates displayed similar gene knockdown efficiencies of TNF-α than unmodified siRNA in the presence of oligofectamine in HeLa cells. In addition, transfection experiments in HuH-7 cells in the absence of oligofectamine demonstrated moderate anti-TNF silencing properties (~25%) at high concentration (100 nM). This indicated that cellular uptake may be favoured by the affinity of the siRNA conjugates containing Gal residues with asialoglycoprotein receptors (ASGPR) [68]. This receptor is highly expressed in mammalian hepatocytes and is responsible for lysosomal processing on *N*-acetylgalactosamine (GalNAc) and Gal residues [69]. Extensive research has been carried out with the synthesis of ASGPR ligand mimics. This has contributed to facilitating cell entry through clathrin-mediated endocytosis [70]. In particular, covalent conjugation of siRNA oligonucleotides with different architectures of GalNAc moieties has resulted in obtaining novel siRNA conjugates with excellent stabilities in the bloodstream and high gene silencing levels in the liver both in vitro and in vivo (Figure 2). These promising results have come to the forefront in pre- and clinical proof of concept [71,72]. Nair et al. prepared several CPG solid supports modified with protected bi-(GalNAc_2_) and triantennary GalNAc residues (GalNAc_3_) [73]. These CPG supports were fully compatible with ODN solid-phase synthesis and allowed them to synthesize series of siRNA-(GalNAc) conjugates targeting both apolipoprotein B100 (ApoB100) [74] and mouse transthyretin (mTTR) [75]. Interestingly, the authors observed great impact on cellular uptake when siRNA passenger strands were conjugated with triantennary GalNAc residues and this was corroborated by high gene silencing levels in vitro targeting the ApoB100 gene in the liver. Furthermore, in vivo experiments confirmed that the presence of the triantennary residue showed efficient liver-specific uptake in mice in the absence of additional transfection agents (Figure 2A). Importantly, subcutaneous administration produced gene expression inhibitions with a single-dose of 1 mg/kg with long-term silencing activities over 9 months without causing any damage and toxic effects in mice.

These results encouraged the same authors to explore the effect of the triantennary GalNAc residues at the 3′-termini but containing sequential GalNAc moieties (up to three residues) through non-nucleosidic linkers [76] (Figure 2B). To accomplish this goal, the authors carried out synthetic strategies that involved the synthesis of CPG solid supports containing three consecutive monovalent GalNAc residues containing, in turn, hydrophobic spacers that may confer an improved recognition of the ASGPR receptor. This was achieved by synthesizing the corresponding pyrrolidine-based CPG solid supports as well as phosphoramidite GalNAc building blocks, respectively. Therefore, a series of siRNA conjugates containing different versions of GalNAc residues were designed to target the same mouse TTR mRNA as described above. In vitro cellular uptake experiments confirmed that siRNA conjugates containing three consecutive GalNAc residues afforded comparable cell entry efficiencies and similar in vivo siRNA potencies than those obtained with the triantennary GalNAc-siRNA conjugate counterpart [73].

Besides obtaining non-nucleosidic building blocks, the same authors carried out structure-activity studies by attaching three consecutive GalNAc residues at the 2′- and 3′-position of the ribosugar and the nucleobase, respectively [77]. This methodology allowed the synthesis of GalNAc-siRNA conjugates targeting mTTR gene by combining “click” chemistry, solid-phase synthesis and phosphoramidite chemistry approaches (Figure 2C). The authors confirmed that three consecutive GalNAc modifications incorporated at the 3′-termini of the siRNA passenger strand did not compromise the RNAi activity, showing similar transfection efficiencies when compared to naked siRNA oligonucleotides. However, when such GalNAc modifications were incorporated at internal positions of the siRNA passenger strand displayed a negative impact on the siRNA potency.

These results highlight that potent silencing activities exhibited by the majority of GalNAc-siRNA conjugates (triantennary and consecutive GalNAc residues, respectively) is determined by modifying the 3′-termini of the siRNA passenger strand and consequently leaving unvaried the 5′-termini of the guide strand [73,76,77]. This is mainly motivated by two factors: (a) Once a synthetic siRNA duplex is introduced into the RISC complex and prior to mediate gene silencing, the siRNA guide strand needs to be phosphorylated at the 5′-termini by a kinase protein hClp1 [78] and (b) The presence of a natural phosphate moiety in the guide strand might be hydrolysed by lysosomal acid phosphatases when GalNAc-siRNA conjugates are administered and therefore reducing their therapeutic efficiency [79].

To increase the stability of phosphate groups at the 5′-termini of the siRNA guide strand, (*E*)-vinylphosphonate (VP) has proven to be a stable phosphate group mimic that has afforded important stability to GalNAc-siRNA conjugates in endocytic processes [80,81]. 5′-VP has shown important biological properties as it is able to prolong the gene silencing efficacy and therefore protect the siRNA from exonucleases (5′-to-3′). Jadhav et al. prepared four GalNAc-siRNA conjugates containing VP groups at the 5′-termini of the guide strand and they were compared to those conjugates containing a 5′-hydroxy (5′-OH) at the guide strand. Additionally, the 5′-VP-guide strand stability was confirmed after incubating and analysing the siRNA duplexes in the presence of lysosomal nucleases under physiological conditions. Interestingly, siRNA conjugates containing the 5′-VP modification showed potent transfection efficiencies in vitro that ranged from 20- to three-fold. This data was also corroborated in vivo in which the authors obtained 3-fold improvement of ApoB silencing after seven days of treatment. These results confirmed the improved metabolic stability of the 5′-VP moiety against 5′-OH counterparts.

Besides promoting the RNAi activity and enhancing the stability of the 5′-VP phosphate mimic in GalNAc-siRNA conjugates when are internalized in vivo, Prakash et al. also increased the chemical diversity by increasing the number of PS modifications in the siRNA duplex [81]. Thus, several siRNA conjugates containing combinations of 5′-VP, 3′-GalNAc with several PS moieties (8 or 16) were prepared to target mouse PTEN mRNA and human apolipoprotein C3 (APOC3) mRNA. The authors found that siRNA conjugates containing 16 PS modifications, the triantennary GalNAc pendent group placed at the 3′-termini of siRNA sense strand and the guide strand modified with the 5′-VP mimic increased the stability and silencing activity of the siRNA conjugate in vitro. These results were corroborated in vivo obtaining 5-10-fold increase in the inhibition of the gene expression in hepatocytes.

**Polymer-siRNA conjugates.** Polymers have become versatile platforms for improving the cellular uptake of nucleic acids and in turn, protecting them against degradation by nucleases [82]. Typically, the electrostatic combination among negatively charged oligonucleotides and cationic polymers results in the formation of polyplexes which are reportedly efficient in a good number of processes like endosomal escape, nuclear uptake and cytosolic transport. These properties have allowed polymer-based carriers to facilitate the cellular internalization of nucleic acids however toxicity and other issues have reduced their potential to be transferred to the clinic [83]. The stability of the polyplexes is an important bottleneck which can depend on the charge density and flexibility of the polymers [84,85]. The improvement of this stability is crucial in order to improve the delivery of small drugs and nucleic acid therapeutics and therefore increasing their therapeutic effect [82,86].

**Figure 3:**
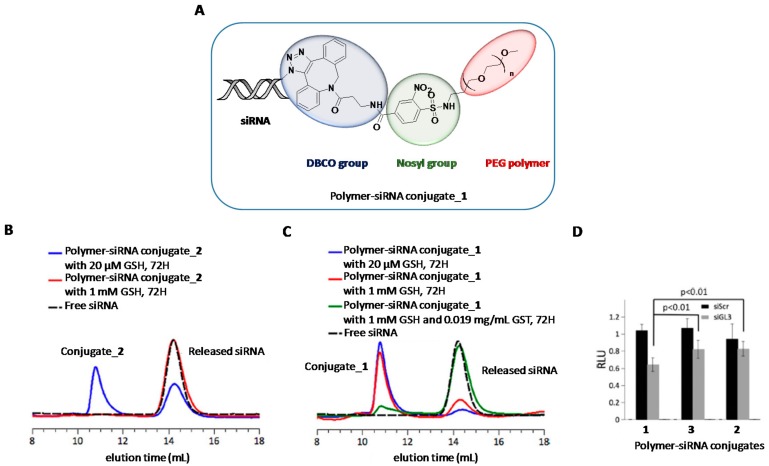
(**A**) Chemical structure of polymer-siRNA conjugate_**1**. (**B** and **C**) Polymer-siRNA conjugate behaviours under extracellular and intracellular environments. SiRNA conjugate containing a PEG disulphide group (conjugate_**2**) liberated a siRNA molecule under extracellular conditions (**B**) whereas the nosyl group (conjugate_**1**) was able to liberate efficiently a siRNA molecule under intracellular conditions (combination of GSH and GST). (D) Gene silencing studies in the presence of lipofectamine in which shows the efficiency of conjugate_**1** (containing the reducible nosyl group) to reduce significantly the production of luciferase. Adapted with permission from ref. [89]. Copyright 2017, Royal Society of Chemistry.

The presence of covalent bonds between nucleic acids and polymers as well as using polymer crosslinking strategies have resulted in the most used strategies for obtaining stable polymer-nucleic acid conjugates [87,88]. In this regard, “click” chemistry and the use of disulphide bonds have become the two well-known strategies that have been used for conjugating polymers directly to siRNA oligonucleotides. As opposed to polyplexes, the correct design of degradable spacers is crucial not only because it has to maintain some stability to resist extracellular milieu but also to be labile upon imparting cellular uptake and thus releasing the cargo. Recently, Huang et al. reported the nosyl group (2-nitrobenzenesulfonamide) as an appropriate linker for polymer-siRNA constructs [89] (Figure 3A). It exhibits resistance to acid and basic conditions and has the special feature of being cleaved by thiol moieties. To confirm this property, the aforementioned cleavable nosyl group was functionalized with a PEG polymer in the first place and secondly with dibenzocyclooctyne-amine (DBCO). This DBCO group has been widely exploited in bioconjugation reactions between alkynes and modified oligonucleotides containing azide groups [90]. Thus, this covalent strategy allowed the authors to prepare novel polymer-siRNA constructs (conjugate_**1**) with potential to respond under intracellular conditions. Additionally, the authors prepared a conventional disulphide linker covalently linked to a siRNA molecule (conjugate_**2**) for comparison purposes. The amount of siRNA release from conjugate_**1** and _**2** was investigated both in extracellular milieu (20 μM GSH, pH 7.4) and intracellular environments (1 mM GSH, +/− GST, pH 7.4) (Figure 3B,C). Interestingly, the authors confirmed the high stability of the nosyl group (conjugate_**1**) as practically inhibited the siRNA release (8%) under extracellular reductive conditions whereas the siRNA containing the disulphide group (conjugate_**2**) displayed a considerable siRNA release (38%) at the same experimental conditions. When GSH concentration was increased up to 1 mM, the integrity of conjugate_**1** was scarcely affected (20% of siRNA release). Finally, total siRNA release was achieved when glutathione S-transferase was added to the GSH solution mimicking the conditions of intracellular environments. To further investigate the potential and stability of this polymer-siRNA construct, the authors carried out gene silencing studies by incubating three siRNA conjugates (one of them containing a non-hydrolysable linker, conjugate_**3**) in the presence of lipofectamine. The authors found significant inhibition activities for conjugate_**1** which reduced luciferase expression up to 40% whereas gene knockdown efficiencies were halved in the other constructs (Figure 3D).

In a related topic, Takemoto et al. engineered an acid pH-responsive polymer-siRNA conjugate capable of enhancing siRNA delivery [91]. The authors used a biocompatible positively-charged polyaspartamide derivative (PAsp(DET)) [92] as a building block to graft siRNA oligonucleotides through “click” chemistry protocols by using a DBCO group and the corresponding azide siRNA [89]. The authors incorporated maleic acid amide (MAA), an acid-labile moiety at endosomal pH but stable at extracellular pH environments [93] between the PAsp(DET) polymer and siRNA. The biodegradability of MAA linker was confirmed by PAGE analysis after incubation at pH 5.0. For comparison purposes, the DBCO-siRNA conjugate was directly conjugated onto the PAsp(DET) polymer in order to achieve polymer-siRNA conjugates without containing non-cleavable linkers. Furthermore, the corresponding polymer-siRNA conjugates were electrostatically combined with Asp(DET), taking advantage of its capacity for enhancing endosomal escape. Cellular uptake experiments were performed and showed an enhancement of 30% in fluorescence when compared to unmodified siRNA polyplex. Gene silencing studies also confirmed that polymer-siRNA conjugates forming polyplexes were able to knockdown luciferase expression more efficiently than control siRNA polyplexes. This inhibition was significantly stronger in the case of polymers containing the MAA linker than non-cleavable functionalities, as expected.

Other polymers with endosomolytic capabilities have been proven to be efficient vehicles for siRNA delivery. Poly(amido amine) (PAA)-based polymer derivatives [94,95] containing cationic amine pendent groups were modified with MAA and poly(ethylene glycol), respectively as amine-masking groups in order to reduce their apparent toxicities. These polymers were easily tuned by introducing additional functional groups to increase their abilities to promote cellular uptake and red blood cell (RBC) lysis activity. PAA polymers were firstly functionalized with an imidazole residue in order to favour the proton-sponge mechanism [96]. Hydrophobic lipid tails and the triantennary GalNAc residue were also introduced to increase the solubility of the polymer and recognize the ASGR receptor, respectively [94,95]. The strategy for introducing the therapeutic siRNA was based on using reducible disulphide bonds between the PAA polymers and the corresponding siRNA oligonucleotide. This strategy resulted in favouring their selective release under high concentrations of GSH inside cells. Structure-activity relationship (SAR) allowed the authors to prepare and identify PAA-siRNA conjugates with improved RBC lysis activities and efficient silencing activities of ApoB mRNA in hepatocyte cells. According to these results, the authors addressed in vivo studies with the most efficient siRNA-polymers obtained in their previous studies. This strategy allowed the authors to obtain potent therapeutic systems with good lytic activities and excellent silencing activities (~60%). In light of these results, a good number of strategies have been accomplished to improve biodegradability rates in vivo and therefore reduce the apparent cytotoxicity of cationic polymers without affecting the silencing activity. These strategies have been focused on introducing additional moieties to the PAA polymer and can be summarized as follows: (i) Derivatization with disulphide linkers [95]; (ii) The use of mixtures of cystamine bis-acrylamide [94]; (iii) Introduction of polypeptides based on a mixture of l-ornithine and l-phenylalanine (in a ratio of 4:1) [97] and (iv) The use of post-polymerization approaches with the aim of modifying siRNA-polymer conjugates with acetic acid, heterocycles with low pK_a_ and acid cleavable acetal linkers [98]. 

Hybrid micelles based on a mixture of a siRNA covalently conjugated to hydrophobic poly(d,l-lactic-*co*-glycolic acid) (PLGA) and linear polyethyleneimine (LPEI) have been also reported [99]. In particular, Lee et al. engineered the corresponding conjugates via a cleavable disulphide linker. This approach was achieved by reacting a thiolated siRNA modified at the 3′-passenger strand, with an activated PLGA which was activated with the heterobifunctional linker 3-(2-pyridyldithio)-propionyl hydrazide (PDPH). This conjugation step between PLGA and a siRNA directed against GFP gene, produced spontaneously the formation of self-assembled micelles in aqueous solution. This process was previously observed by the authors when conjugated PLGA polymer with an antisense oligonucleotide [100]. The resultant siRNA conjugate was electrostatically coated with two LPEI polymers of different molecular weight (25,000 and 2500 Da) in order to generate positively-charged siRNA conjugates with average sizes of 30 nm. Cellular uptake and in vitro gene silencing studies mediated by PEI/siRNA-polymer were performed at several N/P ratios. The authors found excellent inhibition activities without affecting cellular viabilities when hybrid siRNA polyplexes were formulated at high N/P ratios with both PEI polymers. This silencing activity was confirmed after analysing the relative GFP mRNA expression by RT-PCR (43 and 53% of inhibition for LPEI 25,000 and 2500, respectively).

Recently, the same authors used LPEI to stabilize PEG-siRNA-polycaprolactone (PCL) self-assembled micelles for increasing the delivery of siRNA oligonucleotides [101]. Related to this, the authors modified the 3′-termini of both siRNA strands. Thus, in the case of the sense strand, the thiolated RNA was covalently linked to an activated PEG polymer with succinimidyl-3-(2-pyridyldithio)propionate (SPDP) whereas the heterofunctional PDPH crosslinker was used to activate PCL and thus facilitate the incorporation of the antisense thiolated RNA. The combination of both modified strands resulted in the formation of the corresponding siRNA-polymer forming stable micelles (PEG-siRNA-PCL). After confirming the efficiency of this system to deliver efficiently a siRNA targeting GFP; Lee et al. proposed to combine Paclitaxel (PCX), a small anticancer drug, with the preformed siRNA micelles (relative molar ratio of 1:4; siRNA:PCX) with the aim of improving the therapeutic efficacy in chemotherapy by combining a small anti-cancer drug and an anti-apoptotic Bcl-2-specific siRNA. This combination generated particles with average sizes of 38 nm. LPEI was finally incorporated into the aforementioned siRNA conjugate containing PCX resulting in a stabilization of the drug delivery system through electrostatic interactions at N/P ratio of 40. The presence of both Bcl-2 siRNA and PCX produced an important anticancer effect and was superior than activities mediated by free PCX, PEG-siRNA-PCL and siRNA-LPEI (up to 1.5- and 2- and 4-fold, respectively) according to caspase-3 activity analysis. Additionally, the synergetic or dual effect mediated by the siRNA and PCX was also confirmed with mitochondrial activity which showed cellular viabilities around 17.6% after four days of transfection when PCX/PEG-siRNA-PCL/LPEI hybrid system was used.

**Lipid-siRNA conjugates.** Lipids are also one of the most used modifications that have been used for being covalently introduced to nucleic acids. For example, bile and fatty acids, cholesterol and a good number of hydrophobic residues have become the main hydrophobic residues to improve important properties of nucleic acids (e.g., cellular uptake, silencing activity efficiencies, stability in the bloodstream and other pharmacokinetic profiles) [102,103,104].

Cholesterol (Chol) has been one of the first hydrophobic residues that has been used in RNA interference with the aim of modifying the 3′- or 5′-termini of a siRNA passenger strand to increase the transfection capacity of siRNAs [103,104]. Recently, Khvorova et al. have described the preparation of modified siRNAs with Chol targeting peptidylprolyl isomerase B, PPIB) and huntingtin, HTT [105,106]. In particular, Chol and a triethylene glycol spacer (TEG) moieties were covalently linked to the siRNA passenger strand at the 5′-termini. This strategy allowed the authors to prepare a library of 94 Chol-siRNA conjugates targeting the human huntingtin gene [105]. Thus, the resultant conjugates were designed not only to maximize stability, biodistribution but also minimize innate immune response. The most potent Chol-siRNA conjugate was selected after screening the transfection potency in vitro. The authors observed that some siRNAs modified with Chol were able to promote robust cellular uptake into primary neurons as well as inducing potent silencing activities of the Huntingtin mRNA expression without using transfecting agents. Interestingly, the most promising Chol-siRNA candidate showed comparable inhibition activities than the unmodified siRNA (4 and 13 pmol/L, respectively). Preliminary in vivo experiments in mouse brain showed important silencing of the Htt mRNA expression in two brain areas where Huntington disease is mainly affected (i.e., cortex and ipsilateral striatum) without significant neuronal toxicities but with an important gradient of diffusion.

Further studies and following similar synthetic strategies, the same authors evaluated the efficacy of Chol-siRNA conjugates to reduce PPIB and Htt mRNA expressions by adding a 5′-phosphonate (5′-VP) moiety at 5′-termini of the guide strand [106]. As described in previous sections, the introduction of non-natural phosphates at the 5′-termini of the guide strand has proven to be beneficial in RNAi mechanism. This was also confirmed by Haraszti et al. [106]. The authors modified the 5′-termini of the guide strand with 5′-VP in order to preserve the stability against phosphatases. Increased silencing activities in vitro of siRNA-Chol conjugates containing 5′-VP (~3-fold higher) were found when compared to free 5′-hydroxyl-siRNA conjugates. However, this improved stability of the siRNA guide strand containing 5′-VP produced a lengthening in the silencing activity in vivo (0.9-3.3-fold higher) and also produced drug accumulation in several tissues like liver, heart and kidneys.

The development of new delivery strategies has helped to improve efficiently the ability of siRNA-Chol conjugates to reach specific tissues and organs in vivo. SNALP and iNOP platforms are part of these technologies which are based on lipid-based systems and generally used as vehicles for improving the delivery of siRNA molecules into the liver [107,108]. The Dynamic Polyconjugate (DPC) platform is another strategy employed for increasing the silencing activity of siRNA therapeutics. Unlike lipid nanoparticle formulations, DPC technology was originally based on the covalent incorporation of a siRNA molecule through cleavable disulphide linker into a synthetic polymer which was modified with a carboxy dimethylmaleic anhydride (CDM) residue and GalNAc targeting ligands [109] as previously described in the previous section [94,95]. Subsequent generations of DPC platforms developed by Arrowhead Research Corporation have been involved in the co-injection of a siRNA-Chol conjugate with a melittin-like peptide containing GalNAc residues. This strategy, designed for the treatment of Chronic Hepatitis B Virus infection (HBV), immensely improved siRNA activity 500-fold when compared to non-encapsulated siRNA-Chol counterpart [110]. These results allowed the company to move both DPC technology and its siRNA candidate (ARC-520) to the clinic [111,112]. However, despite promising therapeutic results obtained in a good number of experiments, this drug was finally withdrawn in Phase 2 by the company (ID: NCT02065336).

In addition to using phosphoramidites for introducing Chol residues at the 5′-termini, several strategies have been described for the introduction of hydrophobic lipid derivatives at the 3′-termini. For example, the use of a pyrrolidine linker was selected to incorporate Chol at the 3′-termini of the siRNA sense strand [103]. This resulted in the preparation of siRNA-Chol conjugates which displayed similar silencing activities in vitro when compared to unmodified siRNAs. This strategy also confirmed reduction in ApoB mRNA levels in liver and jejunum when siRNA-Chol conjugates were intravenously administered in mice (57% and 36% for apoB-1 and apoB-2, respectively). In light of these findings, the 5′-termini of both siRNA strands was also explored by screening three series of hydrophobic phosphoramidite derivatives based on Chol, litocholic acid and 12-hydroxy lauric [104]. The corresponding modified siRNAs were designed for inhibiting *E. coli* β-galactosidase mRNA. These studies confirmed that Chol hydrophobic residues introduced at the 5′-termini of the sense strand showed superior knockdown efficiencies when compared with the rest of the siRNA modifications and unmodified siRNA molecules. These potent silencing activities in vivo and cellular uptake mechanism were further characterized [113]. Wolfrum et al. demonstrated that some lipoproteins were involved in the internalization and the improvement of the gene silencing activity (e.g., Chol-siRNA and other conjugates modified with fatty and bile acids). In particular, the authors found that association of either high-density lipoprotein (HDL) or low-density lipoprotein (LDL) with Chol-siRNA conjugates was able to promote selectively tissue accumulations. Thus, siRNA conjugates were accumulated in liver, gut and kidney when were bounded to HDL lipoproteins, whereas in the case of LDL lipoproteins, this siRNA accumulation was mainly found in liver. This accumulation mediated by HDL was significantly higher than those obtained with free siRNA-Chol conjugates. Subsequent studies showed that HDL particles were able to be taken up by cells though SR-B1 receptor-mediated mechanisms [114].

Taking the effect of HDL into consideration on gene silencing and transport of macromolecules, Ding et al. found promising in vitro and in vivo activities of siRNA-Chol against hepatocellular carcinoma (HCC), which is one of the most common cancer disease worldwide [115]. Specifically, the authors engineered particles made of reconstituted HDL (rHDL) and siRNA-Chol conjugates (rHDL/siRNA-Chol) designed to knockdown erythroid myeloid ontogenic factor [116]. The corresponding nanocomplexes were characterized by atomic force microscopy (AFM) and dynamic light scattering (DLS) obtaining average sizes of ~60 nm with good encapsulation efficiencies. These hybrid nanoparticles demonstrated comparable cellular uptake efficiencies in vitro (50%) than those carried out with commercially available transfecting agents (~55%). Furthermore, efficient decrease in Bcl-2 protein downregulation and significant inhibition of the cellular growth in HepG2 cell lines were found when targeted siRNA-Chol conjugates forming complexes with rHDL lipoprotein were used if compared to non-targeted siRNA controls. In vivo experiments confirmed that such nanoparticles were accumulated into the tumour and reduced the inhibition of tumour growth and the Bcl-2 expression.

**Figure 4:**
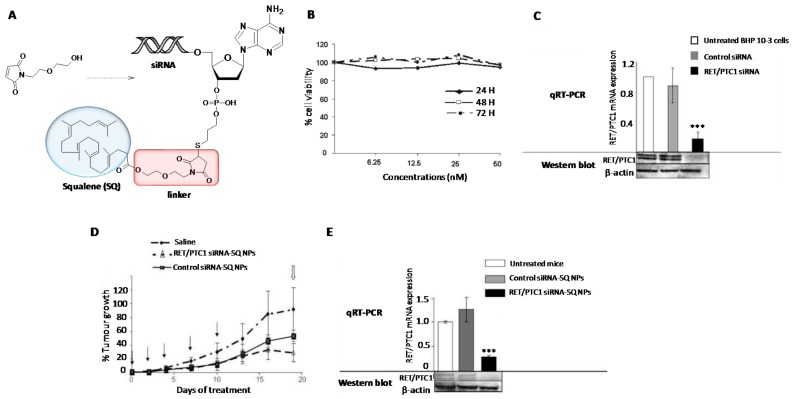
(**A**) Synthetic strategy for the preparation of SQ-siRNA NPs. (**B**) MTT assay at several concentrations of SQ-siRNA NPs and incubation times. (**C**) Reduction of RET/PTC1 mRNA expression in vitro after incubating siRNA RET/PTC1 (60 nM) in the presence of lipofectamine. (**D**) Effect of SQ-siRNA NPs in reducing the tumour growth after 20 days of treatment. (**E**) Reduction of RET/PTC21 mRNA expression measured by qRT-PCR after incubation with SQ-siRNA NPs in vivo. The silence activity mediated by SQ-siRNA NPs was significant (*) when compared to untreated cells according to ANOVA analysis. A scrambled siRNA sequence (control siRNA) was used in order to determine the specificity of the gene silencing process. Adapted with permission from ref. [118]. Copyright 2011, American Chemical Society.

In addition to Chol, other natural products have been covalently conjugated into siRNAs [117] and nucleoside analogues for the development of potent nanomedicines against cancer [118]. Squalene (SQ) is a naturally occurring isoprenoid hydrophobic compound that takes part in the synthesis of Chol as an intermediate. Similar to Chol, SQ can be loaded and distributed to other tissues in association with LDL lipoproteins [119]. Raouane et al. proposed to increase the nucleic acid lipophilicity by covalently linking a SQ residue with a siRNA targeting the junction oncogene RET/PTC1 [118] (Figure 4). This combination resulted in increasing the stability in the bloodstream as well as improving the internalization properties of the siRNA conjugate. Chemically, the introduction of a SQ moiety at the 3′-termini of a siRNA passenger strand is not trivial. The authors were successful when SQ was activated with a maleimido group but intercalating an ethoxyethanol linker between the activating group and SQ [118]. The final conjugation between a thiolated siRNA and the anticipated SQ-maleimide linker was achieved by microwave irradiation with good yields (55%) (Figure 4A). The resultant SQ-siRNA conjugate was able to self-assemble in aqueous buffer leading to modified nanoparticles with average sizes of 165 nm, according to DLS measurements. After confirming the stability of the SQ-siRNA conjugate in serum and its lack of toxicity in cells (Figure 4B), in vitro and in vivo transfection experiments confirmed the efficient knockdown of the RET/PTC1 mRNA in the presence of commercially available cationic lipids (~80%) (Figure 4C) and the ability of SQ-siRNA nanoparticles for inhibiting the tumour growth (~70%) in model mice xenografted RET/PTC1 (Figure 4D,E).

α-tocopherol (TP) is a natural hydrocarbonated molecule that has been explored as a suitable carrier for siRNAs. As described with Chol and other natural compounds, TP has a tendency to be transferred to LDL lipoproteins in the liver [120]. In this article, Nishina et al. modified the 5′-termini of a siRNA guide strand through phosphoramidite chemistry taking advantage of the hydroxyl group placed at the C6 position of the TP. The authors confirmed that the TP modification afforded much higher stability to siRNA than the unmodified counterpart when incubated during 24 h. Furthermore, transfection experiment results in the presence and in the absence of a transfecting agent indicated that the design of asymmetric siRNA conjugates containing the TP modification at different positions did not have a negative impact on the RISC machinery. After one single injection, the reduction of ApoB levels was maximal on the first day of treatment in vivo. Interestingly, TP-siRNA conjugate was able to promote the knockdown of ApoB expression in a dose-response manner using 2, 8 and 32 mg/kg of modified siRNA. To increase the effectiveness of this therapeutic approach in vivo, the authors formulated the same asymmetric TP-siRNA conjugate in a mixture of PEG-60 and linoleic acid to give rise to the corresponding lipid nanoparticles [121]. The authors characterized the cellular uptake process mediated by the TP-siRNA nanoparticles and found that the modified TP-siRNA conjugate was able to bind to chylomicrons and finally transported to the systemic circulation through the lymphatic route. Consequently, the TP-siRNA conjugate was taken up by liver hepatocytes which caused a reduction in ApoB mRNA expression and also was able to reduce low-density lipoprotein Chol as well as triglycerides serum levels. Further studies have demonstrated the affinity of TP to HDL lipoproteins. This effective binding with TP-siRNA conjugate has allowed to explore new therapeutic approaches for reducing BACE1 (β-site AβPP cleaving enzyme 1) protein expression which has been found up-regulated in Alzheimer disease. In vitro and in vivo results suggested the presence of lipoprotein receptors which increased the cellular uptake of the corresponding siRNA in brain and therefore enhanced the expected gene silencing effect [122].

Other small molecules have been described as scaffolds for introducing chemically modified siRNAs at the 3′-termini. Thus, Grijalvo et al. used a protected form of glycerol for modifying the passenger strand with hydrophobic residues containing neutral and cationic lipids [123,124]. The authors prepared a small library of siRNA containing hydrophobic residues (single and double lipid alkyl chains) both at the 3′- and 5′-termini of the siRNA passenger strand. The resultant siRNA conjugates were designed for reducing TNF-α levels in vitro. These authors found that hydrophobic siRNA conjugates achieved comparable silencing activities than unmodified siRNA in the presence of a transfecting agent. Interestingly, transfection experiments in the absence of commercially available cationic lipids showed that modified siRNAs containing a double-lipid saturated alkyl chain at the 5′-termini displayed at least 2-fold increase in the silencing of the TNF-α production than those siRNAs modified with a single linear lipid tail at the 3′-termini According to these results, Ugarte-Uribe et al. characterized cell-entry mechanisms mediated by a oligodeoxynucleotide containing the same double-lipid tail modification at the 5′-end [125]. Internalization studies were carried out in several tumour cell lines. The authors showed that cellular uptake process mediated by the double-lipid modification was enhanced for those cells lines expressing β_2_ integrin (CR3). However, this cellular uptake was less efficient in the presence of CR3-receptor knockout cells.

**Figure 5:**
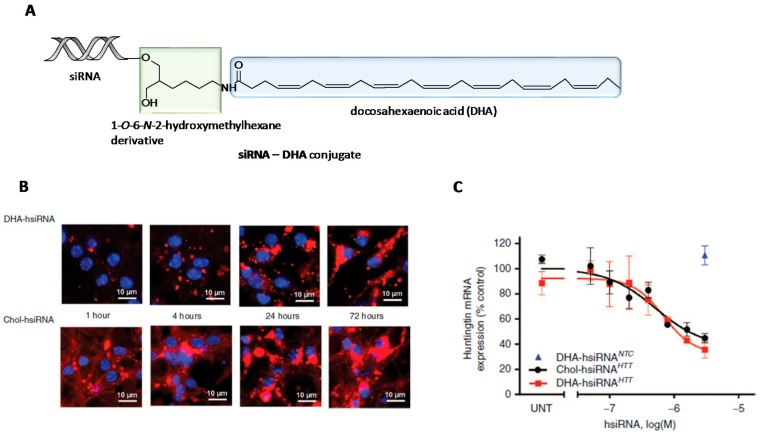
(**A**) Synthetic strategy for the preparation of L-serine-based siRNA conjugates (green) modified with docosahexaenoic acid (DHA) (blue) at the 3’-termini of the passenger strand. (**B**) Confocal images at various incubation times (1, 4, 24 and 72 h) when DHA-siRNA and Chol-siRNA conjugates were incubated in the presence of neurons. (**C**) Reduction of *Mtt* mRNA expression mediated by DHA-siRNA and Chol-siRNA conjugates. Adapted with permission from ref. [126]. Copyright 2016, Nature Publishing Group.

The continuing interest on the search of a therapeutic platform for the treatment of neurodegenerative diseases allowed Nikan et al. to derivatize 1-*O*-DMT-6-*N*-Fmoc-2-hydroxy-methylhexane with docosahexaenoic acid (DHA) [126] (Figure 5). This fatty acid is one of the main components of human brain and is also implicated in some memory-related functions [127]. The resultant fatty acid derivative was directly attached to the control-pore solid (CPG) support which allowed the synthesis of siRNA conjugates containing DHA at the 3′-termini of the siRNA sense strand (siRNA-DHA conjugate) (Figure 5A). The resultant lipid-siRNA conjugates were designed for targeting both Huntingtin (*Htt*) mRNA and CyclophilinB, a binding protein that is involved in some neurodegenerative disorders [128]. To determine the effect of DHA modification, the authors carried out parallel studies by comparing DHA-siRNA conjugate with a siRNA modified with Chol. These results confirmed that DHA-siRNA conjugates promoted cellular uptake in neurons with comparable accumulations than Chol-siRNA conjugate after 72 h incubation (Figure 5B). This effect was also reflected in a decrease in *Htt* mRNA expression (Figure 5C). In vivo studies involving DHA-siRNA conjugate revealed siRNA retention in *ipsilateral stratium* and *cortex* after one-single intrastriatal injection. Interestingly, Htt mRNA expression was significant reduced after one week in both tissues where DHA-siRNA conjugates were accumulated (i.e., 73 and 51% of inhibition activity in the *ipsilateral stratium* and *cortex*, respectively).

In the case of targeting Cyclophilin B, DHA-siRNA conjugates were able to silence mRNA expression up to 80% without inducing neuronal toxicity and innate immune response. To increase the effect of the siRNA-DHA conjugate in the central nervous system (CNS) distribution but maintaining its lack of toxicity, the same authors proposed to incorporate additional chemical modifications based on lysophosphatidylcholine (LPC) moieties in order to favour siRNA delivery to the brain in vivo [129,130]. The authors designed an elegant synthetic strategy in which involved the CPG solid support functionalization with a l-serine-based phosphocholine derivative containing a DHA pendent group. This strategy allowed to modify the 3′-termini of a siRNA passenger strand, obtaining the corresponding siRNA-DHA-PC conjugates designed for silencing *Htt* mRNA expression in mouse brain. The authors observed that siRNA conjugates containing the combination DHA-PC were able to mediate cellular uptake in mouse primary cortical neurons and also promoted silencing activities of *Htt* mRNA expression (80%) as well as reducing the amount of protein (~71%) in the striatum after one week.

These silencing activities were comparable with the siRNA conjugate only modified with DHA (siRNA-DHA conjugate) but increasing neuronal toxicity and stimulating astrogliosis after a single intrastriatal injection (50 μg of siRNA-DHA-PC).

**Figure 6:**
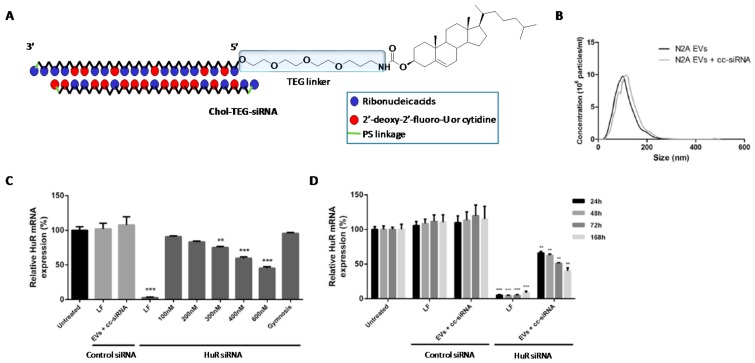
(**A**) Chemical structure of Chol-siRNA conjugate containing the hydrophobic residue at the 5’-termini of the siRNA passenger strand. (**B**) Dynamic light scattering (DLS) of EVs alone and in the presence of Chol-siRNA conjugate. (**C**) Dose-response at several concentrations of EVs:siRNA (100, 200, 300, 400 and 600 nM) targeting HuR mRNA expression. (**D**) Evaluation of silencing process during time (24, 48, 72 and 168 h). * represents significant values of Chol-siRNA conjugate when compared to negative controls (untreated cells). Adapted with permission from ref. [133]. Copyright 2017, The American Society of Gene and Cell Therapy.

**Extracellular vesicles.** In addition to LDL lipoproteins which have become promising non-viral carriers as described before [115], other extracellular vesicles (EVs) have also proved to be efficient nanomaterials for delivering macromolecules [131]. EVs are usually secreted by the majority of cells and have demonstrated an important role in cell-*to*-cell communication process. Depending on size and composition, EVs can be classified into exosomes and microvesicles. Examples of exosomes as carriers for the delivery of siRNA molecules have been previously reported both in vitro and in vivo [132,133]. O’Loughlin et al. used phosphoramidite chemistry to modify the 5′-termini of siRNA passenger strand with Chol and triethylene glycol spacer (TEG) (Figure 6A) [133]. The authors carried out a thoroughly study based on characterizing the encapsulation process between Chol-TEG-siRNA conjugate and EVs secreted from Neuro2a cells. This encapsulation was analysed according to several parameters like dynamic light scattering (DLS), volume, temperature, incubation time and EVs:siRNA ratio (Figure 6B). Optimized conditions (15 molecules of siRNA conjugate per EVLs) were used to promote gene silencing of HuR mRNA expression in HEK293 cells. Interestingly, inhibition activities showed dose-response profiles (Figure 6C). Additionally, the authors assessed gene silencing experiments with optimized conditions at different incubation times and evaluated how the expression of HuR was modulated (Figure 6D). The authors also demonstrated the efficiency of this nanovector in different cells lines (N2A, SH-SY5Y and GM04281 cells) obtaining significant gene silencing activities. These promising results would open new therapeutic approaches for the development of novel cancer-based therapeutics.

**Aptamer-siRNA chimeras.** Aptamers are synthetic oligonucleotides isolated from combinatorial libraries that are able to recognize and bind to specific target molecules with high affinity [134]. Aptamers can have around 20 to 80 bases and are selected in vitro from repeated rounds of large random sequence using SELEX technology [135]. Aptamers have become promising nucleic acids both in diagnostic [136] and therapeutic fields [137]. Thus, aptamers have been designed to recognize cell surface receptors in order to mediate targeted delivery of active therapeutics both in vitro and in vivo. This has allowed to improve the activity of small drugs [137,138] and siRNA [137,139] among others [140].

There are multiples approaches for conjugating siRNAs with aptamers as described by Zhou and Rossi in an excellent review [141]. In this regard, (i) An aptamer can be directly conjugated into the 5′-termini of a siRNA sense strand and finally incubated with the corresponding complementary strand; (ii) Aptamer and siRNA can be conjugated by using a linker moiety which connect both molecules; (iii) Synthesis of “dual-nanoparticles” in which the corresponding aptamer acts as a targeting ligand whereas siRNAs can be incorporated either via thiolated siRNAs or forming complexes. These conjugation strategies can be found on the following research articles: Lupold et al. engineered second-generation aptamer-siRNA chimeras for treating systemic prostate cancer. In particular, the authors engineered three separate compounds; a RNA aptamer like A10-3 which targets a prostate-specific membrane antigen (PSMA) [142] and was electrostatically combined with the sense strand of a DNA-PK-targeting siRNA by a 3′-extended bridge. The corresponding siRNA antisense strand was finally annealed giving rise to the corresponding aptamer-siRNA chimera with high efficiency (~90%). Experiments with Dicer confirmed its selectivity as a substrate processing the DNA-PK targeting siRNA in vitro [143]. This chimera afforded significant stability to siRNA in human and mouse media. The efficiency of silencing DNA-PK in prostate cancer cells was confirmed also in vitro in the absence of a transfecting agent increasing this gene silencing activity via radiation sensitizers. These results were confirmed in vivo following intravenous injections which showed the ability of the chemically A10-3-siRNA chimeras to knockdown specifically DNA-PK in subcutaneous PSMA-positive tumours when compared with aptamer chimeras incubated in the presence of PSMA-negative tumours.

Berezhnoy et al. carried out a detailed study by conjugating 4-1BB aptamer with multiple siRNA targets by using a Durascribe T7 polymerase, which introduced 2′-fluoro-modified pyrimidines in the RNA backbone [144]. The authors designed the corresponding chimeras with a transcription kit by attaching either the siRNA guide strand or passenger strand with the 3′-end of the aptamer. Interestingly, they found improved inhibition activities when siRNA sense strand was conjugated to the aptamer instead of the antisense strand. However, when the same aptamer was co-transcribed with the passenger strand at the 3′-termini but removing its 3′-overhangs, the silencing activities were comparable to unmodified siRNA duplex. By the other hand, such siRNA activity was directly correlated with its melting temperature (T_m_) when conjugated with the 4-1BB aptamer.

Novel aptamer-siRNA chimeras based on bivalent aptamers targeting PSMA were efficiently engineered to evaluate their efficacy for treating prostate cancer in vitro and in vivo (102) [145]. In particular, Liu et al. chose two siRNAs that were designed for targeting both Survivin and EGFR mRNA expression [146,147] and were positioned and covalently linked between the two aptamers. The prepared bivalent aptamer-siRNA chimeras were highly stable when incubated in the presence of fresh human serum up to 24 h and were also recognized by Dicer which resulted in the formation of two 21mer-siRNA duplexes targeting Survivin and EGFR after processing by Dicer. According to in vitro studies, these two siRNAs were able to mediate cellular uptake and inhibit Survivin (~50–60%) and EGFR (~40–50%), simultaneously without using lipofectamine. In vivo experiments confirmed that bifunctional aptamer-siRNA chimeras caused angiogenesis inhibition by EGFR-dependent mechanisms and proved to be efficient therapeutic agent against tumour growth in C4-2 PCa xenograft models.

The rapid evolution experienced in the field of RNA interference has allowed to develop novel strategies to increase siRNA delivery involving nanoparticles [148]. In addition to obtaining beneficial features in therapy, the use of nanoparticles has shown remarkable impact on diagnosis and in vivo imaging [149]. In this regard, quantum dots (QDs) have received special attention [150]. Gao and Bagalkot delved into a study based on aptamer-siRNA chimeras on QDs which not only improved a specific delivery from PSMA-siRNA chimeras but also provided further tools for having a deep insight into their cellular internalization [151]. The authors developed two-step processes to functionalize QDs with PSMA-siRNA chimeras. The success of this technology allowed the authors to evaluate silencing efficiencies of GFP protein and their ability for promoting cellular uptake by covering QDs with poly(maleic anhydride-alt-1-tetradecane, PMAT) and PEI. This design allowed the authors to afford QD-PMAT-PEI nanoparticles with improved stabilities in aqueous solutions and the ability of promoting siRNA escape from endosomes. The incorporation of the chimeras was carried out via thiol-disulphide exchange reaction either by immobilizing directly the PSMA-siRNA chimera or attaching an activated PSMA aptamer onto the QD surface which was previously functionalized with the corresponding siRNA targeting GFP. These aptamer chimeras did not cause any cytotoxicity in cells at 4 and 10 nM, respectively. Interestingly, the authors found distinct QD-PSMA-siRGFP chimera behaviours in PSMA-positive GFP expressing cells. These results were confirmed when siRNA-aptamer chimeras containing the appropriate orientation and better accessibility displayed improved gene silencing efficiencies (~34% more silenced cells) when compared with siRNA chimeras that were directly loaded onto cationic nanoparticles.

Novel architectures based on aptamer-siRNA chimeras have been described for improving receptor-specificity and therefore mediating efficiently cellular uptake via endocytosis [152]. Yoo et al. designed efficient multivalent aptamer-siRNA conjugate for promoting the cellular delivery of siRNAs [153]. Firstly, the authors used a DNA aptamer targeting mucin 1 (MUC1), a protein that is highly expressed on several tumoural cell lines [154]. The antisense siRNA strands were linearly conjugated by using a thiol-maleimide reagent (dithio-*bis*-maleimidoethane, DTME) and this resulted in obtaining either multimeric or dimeric antisense siRNAs. Final hybridization of the resulting antisense siRNA strands with MUC1 aptamer-siRNA chimeras gave rise to the formation of multimeric (Comb-Apt-siRNA) and dimeric (Di-Apt-siRNA) chimeras, respectively. Interestingly, the presence of multiple ligands helped to promote excellent cellular internalizations for Comb-Apt-siRNA in MCF-7 expressing MUC1 cells when compared to fluorescently labelled dimeric and monomeric chimeras with POPO-3 dye. These internalization studies were also carried out in the presence of HepG2 cells (negative control). The authors confirmed that internalization mechanisms were mediated by clathrin-dependent endocytosis. Additionally, cell viability and gene silencing studies targeting GFP and Bcl-2 were also studied. In both cases, Comb-Apt-siRNA was able to mediate gene expression inhibition with better efficiencies than the rest of the prepared aptamers. To facilitate endosome escape and therefore increase the suppression of MCF-7 and HepG2 cell proliferation by inhibiting Bcl-2 mRNA expression, the authors combined the aptamer chimeras with LPEI before transfection experiments. This strategy improved remarkably the ability of multivalent Comb-Apt-siRNA chimeras to mediate siRNA delivery into target cancer cells and therefore reduce the viability in MCF-7 cells. As expected, no significant effect of the constructs was observed in the case of HepG2 cells. In addition to introducing relevant siRNA sequences like Bcl-2, the same authors were able to engineer a dual drug delivery system made of the aforementioned multivalent aptamers but containing an additional intercalated chemotherapeutic drug (Doxorubicin, DOX) [155]. Thus, the construction of multivalent Dox-Apt-siRNA chimeras resulted in reducing significantly cellular viabilities as well as increasing apoptosis levels in multidrug-resistance (MDR) cancer cells.

**Figure 7:**
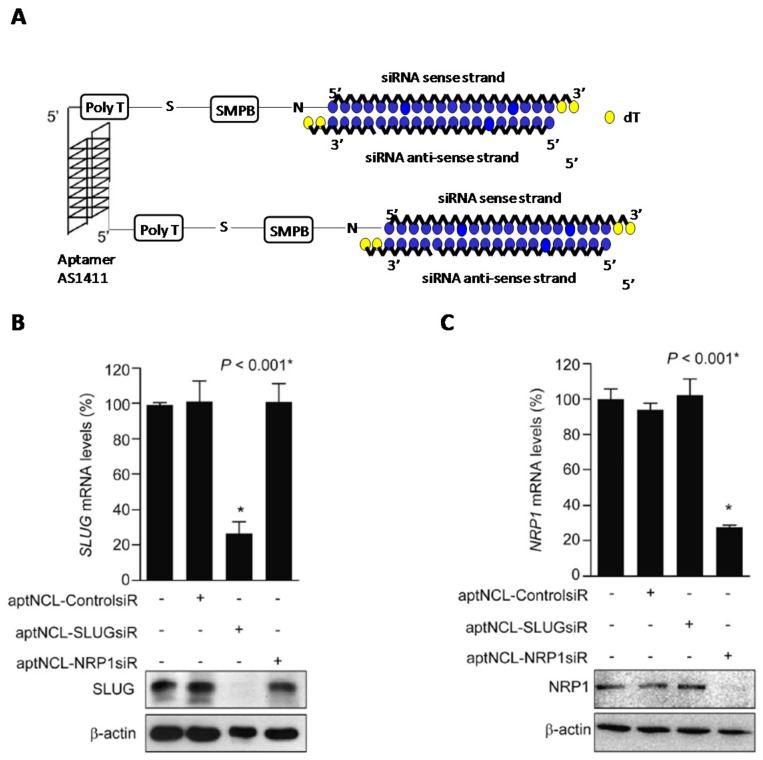
(**A**) Design of aptamer-siRNA chimeras targeting SLUG and NRP1 genes. (**B**,**C**) Inhibition of gene expression mediated by aptamer-siRNA chimeras targeting SLUG (**B**) and NRP1 (**C**) mRNA expressions. * represents significant values of the siRNA nanostructure when compared to untreated cells. Adapted with permission from ref. [156]. Copyright 2014, Elsevier.

Aptamer AS1411 has proved to be an extraordinary targeting ligand for highly cell surface-expressed nucleolin (NCL) in the plasma membrane. This important property has been used to engineer efficient delivery systems to facilitate the internalization of a good number of therapeutic drugs, antisense and siRNA oligonucleotides into cancer cells [156,157,158,159]. Lai et al. were able to construct two aptamer-siRNA chimeras that were used to knockdown SLUG (snail family zinc finger 2) and NRP1 (neuropilin 1) mRNA gene expressions [156] which are directly involved in lung cancer metastasis and angiogenesis (Figure 7). To construct both chimeras, the authors conjugated the 5′-termini of AS1411 aptamer to the 5′-termini of the selected siRNA passenger strands. Both entities—aptamer and siRNA—were separated from one another with a poly(T) spacer by using a *sulfo*-(succinimidyl-4-(*n*-maleimidophenyl)-butyrate; *sulfo*-SMPB) linker (Figure 7A). The authors confirmed the introduction of such linkers did not affect the integrity of the aptamer. In vitro gene silencing studies showed that both chimeras were able to inhibit individually SLUG and NRP1 with high efficiency in NCL-expressing cancer cells (Figure 7B,C). Interestingly, the authors also assessed combined treatments with the two aptamer-siRNA chimeras demonstrating the synergic effect when mixed both aptamer chimeras. The silencing of both SLUG and NRP1 mRNA expression was simultaneously inhibited producing a larger reduction in cell motility (20.6%) and cell invasion (34.6%). These promising results were confirmed in vivo using xenograft mouse lung cancer models. To confirm the potential of the combined therapy, authors inoculated subcutaneously the single chimeras and, on the other hand, the combination of both chimeras. Interestingly, the combined treatment caused a 4-fold reduction of the tumour growth whereas in the case of single chimera treatment produced a 3-fold reduction when compared to aptamer controls. Furthermore, the synergetic effect of the combined-chimera treatment reduced more efficiently lung cancer invasion and angiogenesis (1.2-fold vs. aptamer control). These promising results have revealed the combined treatment of aptamer-siRNA chimeras may be a suitable approach for targeted cancer chemotherapy treatments.

## 3. Chemical Modifications and miRNA Delivery

MiRNA molecules are often expressed in a good number of cells and tissues [160] and also play a pivotal role in the regulation of various biological processes like proliferation and apoptosis, among others [161]. In that way, the evidence shows that appearance of cancer development states can be caused by the expression of aberrant miRNAs which act as oncogenes and tumour suppressors. Lin-4 was the first miRNA which was discovered in the early 1990s in *C. elegans* [162]. Ten years later, a second miRNA (let-7) was characterized in the same organism and also identified in humans [163]. Since then a large number of miRNAs has been identified along with their biologic function. A representative list of miRNAs is displayed on Table 2.

The process of miRNA regulation has become an important issue for the discovery of new drugs in last years. A good number of approaches such as the synthesis of “*antagomiRs*” (anti-miRs), locked nucleic acids (LNAs), peptide nucleic acids (PNAs), which are able to interact specifically with target oncogenic miRNAs as well as the introduction of miRNAs or miRNA mimics in order to reconstitute tumour suppressor activity have been opened up novel pathways for the search of alternative cancer-based therapies [164,165,166].

**AntagomiR conjugates**. Synthetic ASOs have proved to be effective molecules for targeting ncRNAs confirming their practicability of regulating miRNAs in vitro and in vivo [24]. This ability of recognition has been observed for miR-122 which is abundantly found in liver and acts as a regulator in several physiological processes involving fatty acid metabolism as well as cholesterol accumulation [167]. By virtue of its biologic properties, miRNA-122 has been selected as an important target for cancer therapy, in particular, hepatocellular carcinomas (HCCs).

The first antagomiR conjugate containing 2′-*O*-methyl modified RNA bases and phosphorothioate modifications at the two ends of the oligonucleotide chain (3′- and 5′-) was engineered by Krützfeldt et al. in 2007 [168]. Following the same strategy acquired in the field of siRNA delivery, the authors prepared the synthesis of antagomiR conjugate series modified with a cholesterol residue [103] at the 3′-termini containing various oligonucleotide lengths. Thus, antgomiR conjugates targeting miR-122 exhibited excellent nuclease resistant and improved cellular uptake in vitro and in liver tissues; however, their silencing activities tied directly to the length of the oligonucleotide. This optimization allowed the authors to obtain potent miRNA inhibitions from antagomiR conjugates containing lengths greater than 19 base pairs, as well as being modified with 6 phosporothioate groups. Additionally, miR-122 has become a potential target for hepatitis C virus (HCV) due to their great affinity for binding to the HCV genome and promoting the replication of the viral content [169]. Silencing of the miR-122 activity has been achieved by covalently modifying anti-miR-122 conjugates with the triantennary GalNAc pendent group (RG-101) [72]. This antimiR has been one of the few miRNA-based therapeutics that have been proved efficient for patients with chronic HCV infection resulting in a decrease of HCV RNA with various genotypes (1, 3 and 4, respectively) [170].

**Locked nucleic acids (LNAs).** In addition to phosphorothioate conjugates, locked nucleic acids (LNAs) have also shown an important approach for targeting miRNAs because of their extraordinary affinity and specificity to interact with RNA complementary sequences as well as displaying high thermal stabilities [183,184]. The LNAs chemical structure resides in an additional bridge that links both the 2′-oxygen and 4′-carbon positions of the ribose which locks the sugar at the 3′-*endo* conformation. This special feature has allowed LNA technology to be an important tool for antisense gene silencing processes and more recently in the search of miRNA-based therapeutics by designing efficient antagomiRs (LNA-antimiRs) for silencing miRNA expressions both in vitro and in vivo [185,186]. Important therapeutic results were achieved by Elmén et al. who engineered a 15-mer oligonucleotide containing LNAs (>50%) which was entirely modified with phosphorothioate residues targeting miR-122 expression [187]. The authors carried out in vivo experiments both in diet-induce obesity mice models and non-human primates by using intraperitoneal and intravenous injections. After one single injection during three consecutive days, a saline formulation based on LNA-antimiR was able to impart cellular internalization and be accumulated in the cytoplasm of hepatocytes. Furthermore, LNA-antimiR was able to exhibit efficient silencing of miR-122 by forming the corresponding heteroduplex LNA-antimiR:miR-122 which resulted in a controlled decrease of total cholesterol in the liver with an effective dose of 10 mg/kg. Subsequent histopathological analysis after LNA-antimiR injections showed any toxicity in liver and other organs like kidneys after carrying out experiments.

As with antimiRs-based drugs modified with GalNAc in which exhibited potent inhibition activities against HCV, Santaris Pharma developed a 15-mer LNA-antimiR oligonucleotide complementary to miR-122 (Miravirsen) which was only modified with phosphorothioate linkages. This drug showed remarkable in vitro antiviral activity [188] and also generated viral suppression in all HCV genotypes without producing viral resistance in vivo [189]. These promising activities allowed to launch Miravirsen into the clinic [190] which proved to be effective and safe long-term drug when injected intravenously or subcutaneously in primates [189] and human patients [191] with chronic HCV.

The versatility of LNA technology have been also demonstrated by targeting other potential miRNA targets involved in cancer diseases. Nedaeinia et al. carried out antisense gene silencing studies with the aim to find inhibitors for miR-21, which is related to various cancers (e.g., breast, cervix, colon and liver among others) [192]. The authors studied the effect of LNA-antimiR-21 conjugate in human colon adenocarcinoma LS174T cell line in order to find inhibitors for treating colorectal cancer (CRC) [193]. This study describes the LNA-antimiR activity from MTT and Annexin V-FITC/propidium iodide assays at different incubation times. Interestingly, it was found that LNA-antimiR-21 was able to inhibit proliferation of cancerous cells by activating apoptosis and reducing cell invasion.

**Figure 8:**
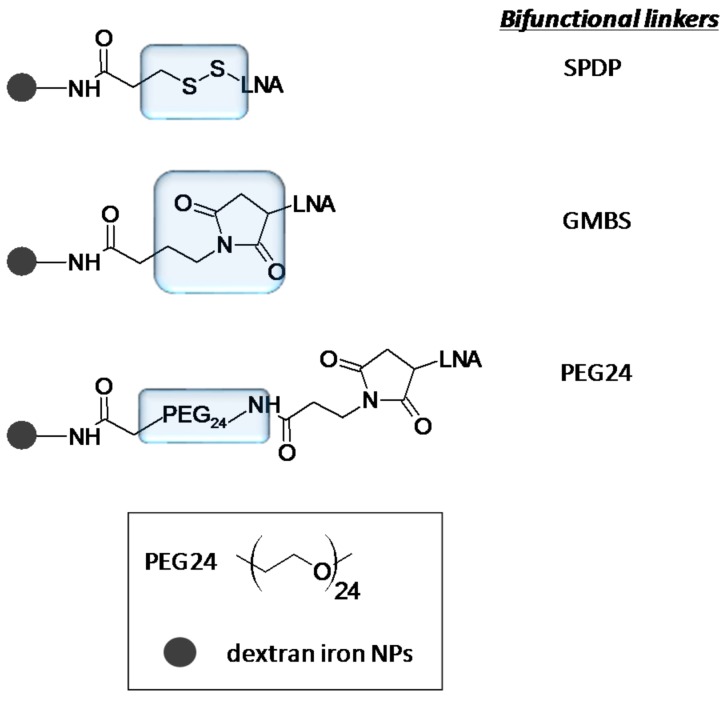
Use of iron NPs functionalized with LNA targeting miR-10b. Structures of three NPs containing SPDP, GMBS and PEG linkers.

The use of nanocarriers comprising both LNA-antimiR conjugates and targeting ligands have allowed Medarova et al. to find a therapeutic approach for inhibiting miR-10b in tumour cell lines [194,195] (Figure 8). This miRNA has been found upregulated in a good number of human cancers like pancreatic, breast and glioblastomas, among others [196]. Furthermore, miR-10b is also involved in metastatic carcinomas although its mechanism of action is still unclear [172]. To reduce the levels of metastasis originating in breast cancer, the authors designed dextran coated iron nanoparticles (NPs) which were successfully functionalized with three different ligands: a fluorescently labelled Cy5.5 dye, a LNA-antimiR-10b conjugate and an integrin receptor RGD peptide (Arg-Gly-Asp) in order to enhance and direct the LNA oligonucleotide delivery into cancer cells. The conjugation of LNA-antimiR-10b and RGD peptide was carried out by using three bifunctional linkers that differed in length and biodegradability properties: (i) SPDP, a short linker which enables dissociation process in intracellular environments in the presence of GSH; (ii) GMBS (*N*-(γ-maleimidobutyryloxy)-succinimide ester, which is a non-degradable short linker and (iii) PEG24, a non-degradable long linker which favours the circulation of NPs without affecting aggregation processes in bloodstream (Figure 8). Interestingly, the authors found that NPs were able to impart cellular uptake almost in their entirely, in particular those NPs containing SPDP and GMBS linkers according to flow cytometry studies. These results were also confirmed by confocal images. Interestingly, only NPs functionalized with the biodegradable SPDP linker were able to inhibit miR-10b expression (63%) when compared to NPs coated with GMBS and PEG24 linkers at the same conditions [194]. This inhibition efficiency was confirmed by flow cytometry and qRT-PCR experiments. Further experiments involving the same design of targeting NPs containing degradable SPDP linker and LNA-antimir-10b were carried out in vivo [195]. Results confirmed the therapeutic action of the miRNA nanoconjugate when was intravenously injected in tumour mice models. Additionally, the authors also showed the LNA nanoconjugate therapeutic potential due to its ability of being accumulated in tumour and lymph nodes and also avoiding metastasis process from being initiated.

**Peptide nucleic acids (PNAs).** In addition to LNAs, peptide nucleic acids (PNAs) have been also used for regulating gene expression [197,198]. They are artificial DNA/RNA molecules in which sugar and phosphate moieties have been replaced by *N*-(2-aminoethyl)glycine residues [199]. Just as in the previous case with LNAs, PNAs are able to recognize complementary DNAs and RNA sequences with high specificity and thus obtaining, through Watson-Crick base pair interactions, the corresponding PNA/DNA and PNA/RNA duplexes, respectively. Taking this high affinity into account, PNA molecules have been built as promising entities for targeting ncRNAs and consequently as a novel approach for developing miRNA-based therapeutics [200]. PNAs are resistant to nucleases and proteases however the levels of PNAs that are internalized in cells are low [201]. To improve this limitation, PNAs have been often covalently modified or complexed with non-viral vectors in order to promote this cellular entry [202,203].

PNAs were used as antimiRs for the first time in 2008, when Fabani and Gait were able to target miR-122 in human and rat liver cells, as a proof of concept [204]. The authors carried out an extensive research based on chemically modifying PNA molecules in order to achieve efficient silencing activities without using transfection agents or electroporation techniques. Thus, the covalent linkage of units such as CPPs (R_6_-Penetratin), four Lys residues and cystamine (Cys) containing terminal-free thiol groups [205] have demonstrated promising covalent strategies for improving cellular uptake through clathrin-dependent and independent routes for achieving the inhibition of target miR-122. The same chemical modifications previously introduced involving Lys residues for targeting miR-155 were used by Fabani et al. [206] This miRNA has proven to have a pivotal role in several biological processes including evolution of malignant growths [207] and the enhancement of cardiovascular disease progression [208]. The authors carried out the transfection of PNA molecules without the help of a transfection agent and also the evaluation of miR-155 levels in vivo by using two intraperitoneal injections at 50 mg/kg. In addition to reducing the expression of miR-155 in splenic B-cells, the authors carried out microarray analyses that confirmed similar gene expression profiles in the case of antimiR-155 and miR-155-knockout-treated mice however this gene profile was entirely found different for wild-type mice.

Other important target that has been currently studied for cancer therapy is the miRNA-221/22 cluster. This miRNA cluster is involved in glioblastoma progression when is upregulated. This produces cell proliferation by targeting PUMA (a p53-upregulated apoptosis modulator) [209] and other processes including cell migration and growth in the case of downregulating tyrosine phosphatase μ (PTPμ) [210]. Furthermore, the upregulation of such cluster has been found in other type of cancers like bladder and breast cancers producing an enhancement of the tumour progression and metastasis [211]. Examples of PNA-based molecules targeting miR-221/222 cluster with the aim to minimize human gliomas have been reported by Brognara et al. by synthesizing a PNA molecule covalently modified with a polyarginine residue (R8) [212,213]. As described before, the presence of such cationic residues facilitates the PNA-antimiR cellular internalization in glioma cells without showing changes in their cellular viabilities. The authors showed that miR-221 expression could be silenced when PNA-antimiR-221 was transfected without affecting the expression of miR-222. This produced the inhibition of cellular proliferation by increasing p27 protein expression. Interestingly the use of PNA-antimiR-221 also produced the upregulation in the expression of another target, TIMP3. The same authors studied the therapeutic effect on glioma cells when combined both PNA-antimiR-221 and PNA-antimiR-222 containing the same R8 residue [212]. Apoptosis levels were analysed and confirmed that combining both PNA-antimiRs addressed against miRNA-221 and 222 were able to induce apoptosis. This produced an increase of the therapeutic effect on glioma cells and therefore suggested a new approach for regulating cell proliferation.

The therapeutic potential of PNAs as antimiR molecules has been also demonstrated for targeting miRNA-210. This miRNA has been found upregulated in several cells and has been involved in cardiac diseases [214], various cancer and some processes related to hypoxia pathways [215]. The miRNA activity has been regulated by using covalent [216,217] and formulation approaches [218]. Covalent approaches were accomplished by solid-phase synthesis which involved either the direct incorporation of an arginine-rich sequence (R8) into the corresponding anti-miR-210 [216] or the use of chiral PNAs (e.g., α- and γ-PNAs) in which the final PNA sequence was made of alternating chiral monomers based on the *N*-(2-aminoethyl)-glycine backbone bearing homo-arginine and arginine side chains, respectively [217]. As observed in previous examples, PNA-antimiR-210 modified with the R8 residue was able to recognize and bind with high efficiency to its corresponding RNA target giving rise to an enhancement in the cellular uptake in leukemic K562 cells after 48 h incubation. This efficient PNA-antimiR internalization generated a whole series of biologic responses which produced a positive impact on silencing the miR-210 activity, the raptor mRNA upregulation along with a decrease of γ-globin.

To avoid undesirable enzymatic degradations and provide fully protection to PNA conjugates from proteolysis, Manicardi et al. proposed to introduce eight cationic residues (Arg) within the PNA backbone containing α- and γ-PNAs without altering the inhibition activity of such PNA-antimiRs. The silencing activity was also evaluated on K562 cells. Interestingly, the new conjugates exhibited resistance when incubated in the presence of serum and peptidases. Furthermore, PNA conjugates were able to impart cellular uptake. This internalization was located entirely in the cytoplasm. Even though all chiral PNA-antimiRs prepared by the authors were able to inhibit the activity of miRNA-210, silencing activities differed according to the modifications introduced into the PNA-antimiR conjugates, obtaining the best inhibition results in the case of γ-PNA-antimiR-210. Although this PNA behaviour is not fully understood, the authors suggested that the presence of side modifications in the PNA backbone might produce differences both in the mechanism of cellular entry of PNA-antimiRs and the recognition with their target miRNA-210 (RNA binding). These important results would allow chemically modified PNA molecules, not only at the end of the sequence but also in their backbone and thus obtaining more potent PNA-antimiR-based drugs.

**Other approaches.** Gold nanoparticles (AuNPs) have provided important contributions as vehicles for transporting small molecule drugs and other kinds of payloads (e.g., proteins, DNA/RNA, antibodies) due to their unique optical properties, stability in the bloodstream and low cytotoxicity [219,220]. Numerous examples have been reported dealing with the use of AuNPs to detect intracellular biomarkers and therefore obtain attractive nanobiosensors for targeting miRNAs [221,222]. The use of AuNPs for delivering miRNAs has been also explored and recently reviewed [223]. The strategy followed to functionalize AuNPs with miRNAs has been assessed by synthesizing the corresponding thiolated miRNAs according to the experience previously acquired in the synthesis of AuNP-DNA conjugates [224]. Hao et al. prepared two AuNP-miRNA conjugates designed to target, in the first instance, PRKCε mRNA by delivering miR-205 and secondly, PTEN (phosphatase and tensin homolog) and E2F1 mRNA by transfecting miR-20a [225]. Both ncRNAs are involved in tumour suppressive and oncogenic roles, respectively. To increase the colloidal stability of AuNPs, the authors modified both miRNA mimics with an hexaethyleneglycol linker (spacer18). Both modified AuNP constructs were able to promote cellular uptake without using commercially available cationic lipids. With respect to the first construct, the authors demonstrated the ability of AuNP-miR-205 to recognize and interact with the 3′-UTR PRKCε mRNA by transfecting pMIR-REPORT vector in HeLa cells. This gave rise to the corresponding duplex formation specifically and confirmed the feasibility of the construct after analysing firefly luciferase activity. To validate these findings, the authors were able to reduce PRKC (protein kinase C epsilon) expression and therefore the cellular survival (~50%) and migration in PCa cells when AuNP-miR-205 was transfected. In the case of oncogenic miR-20a, constructs based on AuNPs-miR-20a also contributed with important activities involved in diminishing both E2F1 and PTEN mRNA expressions (32 and 41%, respectively) by enhancing approximately 3-fold the cellular survival when compared with transfections mediated by commercial cationic lipids.

Other nanoconstructs have been proposed by Kim et al. for targeting miR-29b which has been considered a key for regulating the expression of Mcl-1 protein as well as apoptosis [180]. The strategy was based on functionalizing AuNPs with single-stranded thiolated RNA I oligonucleotides [226]. These AuNP conjugates were annealed with an antisense DNA linked to anti-miR-29b and finally applied to HeLa cells [227]. Interestingly, the authors showed two factors when inhibited miR-29b. Firstly, a significant upregulation of the Mcl-1 protein expression level was observed and secondly, an important apoptosis inhibition promoted by TRAIL (tumour necrosis factor-related apoptosis-inducing ligand) [228] when targeting the 3′-UTR Mcl-1 protein. This strategy was further studied by the same authors with other miRNAs in order to get a reliable platform for delivering anti-miRNAs, in particular miR-21 in both KGN and 293T cell lines.

**Figure 9:**
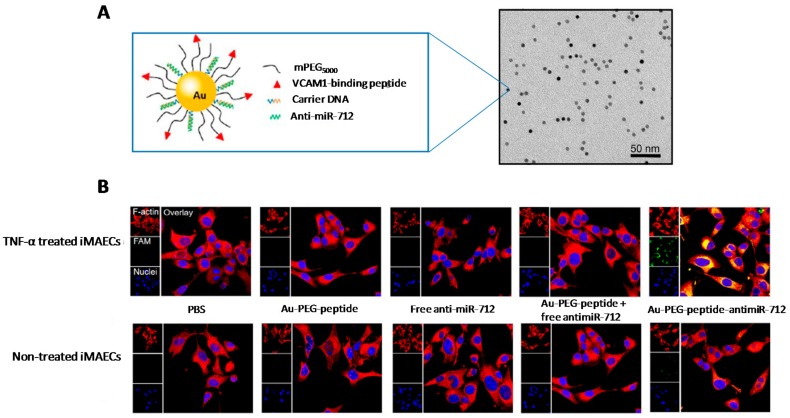
(**A**) Design and a TEM image of the AuNP conjugates decorated with VCAM1 binding peptide and anti-miR-712. (**B**) Confocal images of iMAECs which show the ability of the AuNPs to impart cellular uptake as well as the release of the fluorescently labelled anti-miR-712 and duplex formation with the corresponding target. This effect was not observed in non-expressing VCAM1 endothelial cells. Adapted with permission from [229]. Copyright 2016, Wiley-VCH Verlag GmbH&Co. KGaA, Weinheim.

More recently Xia et al. prepared and functionalized Au nanospheres to be addressed to target miR-712 (Figure 9) [229]. Mechanistic studies have identified the pivotal role of miR-712 in several processes like endothelial inflammation and atherosclerosis [181,182]. Because miR-712 has been found upregulated in such inflammation processes, the authors aimed to deliver anti-miRs for suppressing and restoring the miRNA activity [182]. This therapeutic strategy was achieved by delivering anti-miR-712 from AuNPs. To increase the selectivity of this DDS, the authors decorated their nanospheres with VCAM1 biding peptide as a ligand in order to promote targeted delivery in endothelial cells through ligand-receptor-mediated endocytosis [230]. After coating AuNPs with two pegylated derivatives (e.g., PEG-SH and NH_2_-PEG-SH), a thiolated DNA oligonucleotide with a spacer of 10 adenine bases was also introduced. Finally, the AuNP was decorated with anti-miR-712 which was hybridized with the aforementioned DNA sequence (Figure 9A). The authors used immortalized murine aortic endothelial cells (iMAECs) which were previously treated with TNF-α to promote overexpression of VCAM1. This approach lies in the duplex destabilization by releasing the anti-miR from the AuNP conjugate once the nanosphere was internalized through endocytosis mechanisms followed by the subsequent duplex formation with the upregulated miR-712 found in such endothelial cells. This mechanism was confirmed after labelling the anti-miR-712 with FAM dye obtaining a pronounce fluorescence in the cytoplasm. These results were also corroborated with flow cytometer experiments showing the ability of AuNPs containing a VCAM peptide to mediate delivery of anti-miR-712 in endothelial cells (Figure 9B). The authors also evaluated the bio-distribution profiles of four AuNPs with several sizes (e.g., 5, 10, 20 and 50 nm, respectively). In vivo experiments in atherosclerosis mouse models confirmed AuNPs displayed distinct bio-distribution profiles resulting in a significant accumulation in the left common carotid artery (LCA) for AuNPs with average sizes of 5 nm. These important results demonstrate the potential shown by AuNP nanostructures to recognize efficiently upregulated ncRNAs and their subsequent therapeutic effect in vitro and in vivo.

## 4. Conclusions and Future Directions

The growing interest generated by nucleic acid-based therapies in recent years has allowed the successful launch of six drugs on the market in addition to showing a good number of candidates at different clinical phases [231]. However other promising candidates have not suffered the same fate. Some of these were withdrawn either at phase III trials (e.g., bevasiranib, a siRNA drug targeting vascular endothelial factor (VEGF) developed by Opko Health) or phase II (e.g., AGN211745 (Allergan), PF04523655 (Quark Pharmaceuticals) and ARC-520 (Arrowhead Pharm. Corp.), which developed siRNAs targeting VEGFR1, proangiogenic protein (RTP801) and HBV sequences, respectively). Despite difficulties and undesirable setbacks related to efficacy, great efforts are being made in developing strategies for the efficient administration of nucleic acids in vitro and in vivo in order to circumvent the drawbacks associated to cellular uptake. Formulation strategies based on combining the most appropriate non-viral vector (e.g., lipids, CPPs and polymers among others) along with the progress of the administration methods have partially overcome the limitation when using nucleic acids as a drug.

The emergence of new strategies that enables the covalent linkage between the aforementioned non-viral vectors with oligonucleotides (i.e., ASOs, siRNAs and miRNAs) has helped to improve other kind of parameters such as stability and pharmacokinetic profiles. Disulphide chemistry is one of the most used covalent strategies in which siRNA molecules have been preferentially linked to CPPs, polymers and aptamers. Thus, once transfected, the presence of this cleavable linker under high concentrations of glutathione in tumour cells promotes the cleavage of the linker and therefore releasing the anticipated siRNAs. Alternatively, the versatility afforded by covalent approaches has also allowed the incorporation of tailed-made small molecules into nucleic acids. For example, excellent therapeutic results have been obtained with the synthesis of a series of siRNA conjugates decorated with the triantennary GalNAc residue. Several families containing this residue are found at various clinical statuses, including one (ALN-TTRsc/Revusiran) indicated for the treatment of amyloidotic cardiomyopathies, which is currently found at Phase III of clinical trials. In addition to carbohydrates, important findings have been achieved for the treatment of Huntingtin disease by developing synthetic strategies for attaching lipids, in particular the linkage of docosahexanoic acid with siRNA oligonucleotides.

The use of covalent approaches has allowed miRNA technology to accelerate the process of achieving promising therapeutic molecules. LNAs and PNAs are good examples of how the synthesis of nucleic acid analogues can specifically interact with high affinity with up-regulated miRNA molecules in vitro and in vivo. Promising results in vivo have been also obtained wit anti-miRs covalently modified with Chol and GalNAc pendent groups, confirming the suitability of such modifications in therapies based on nucleic acids. While we still have a long way to go, the possibility of surpassing the bottlenecks associated to gene silencing activity by covalently introducing targeting ligands have resulted in a nice approach for improving cellular internalization and an important strategy for revitalizing antisense, siRNA and miRNA-based therapies.

## Figures and Tables

**Table 1:** Representative nucleic acid-based therapeutics targeting ASOs, siRNAs and miRNAs under clinical trials.

Drug Name	Company	Nucleic Acid	Target	Clinical Trial
Oblimersen	Genta	ASO	Bcl-2	Phase III
Apatorsen	Ionis Pharm	ASO	Hsp27	Phase II
Resten-MP	AVI Biopharm	ASO	c-myc	Phase II
ALN-TTR02	Alnylam	siRNA	TTR	Phase III
Bevasiranib	OPKO Health	siRNA	VEGF	Phase III
SYL040012	Sylentis	siRNA	ADRB2	Phase II
AZD9150	AstraZeneca	siRNA	STAT3	Phase II
PF-655	Quark Pharm	siRNA	RTP801	Phase II
DCR-MYC	Dicerna Pharm	siRNA	c-myc	Phase II
Fitusiran	Alnylam	siRNA	AT3	Phase II
Atu027	Silence Ther	siRNA	PKN3	Phase I
RXI-109	RXi Pharm	siRNA	CTGF	Phase I
ALN-VSP	Alnylam	siRNA	VEGF	Phase I
IMO-2125	Idera	siRNA	TLR9	Phase I
RG-101	Regulus	miRNA	HCV	Phase II
Miravirsen	Roche	miRNA	HCV	Phase II
RG-125	Regulus	miRNA	miR-103/107	Phase I
MRG-106	miRagen	miRNA	miR-155	Phase I
MRX-34	Mirna	miRNA	miR-34	Phase I

**Table 2:** Representative miRNAs involved in cancer diseases and covered in this review.

miRNA	Target	Biologic Function	Reference
Let-7	c-myc, HMGA2, RAS	Tumour suppressor	[163,171]
miR-10b	HOXD10	Oncogene	[172]
miR-21	PTEN, TPM1, PDCD4	Oncogene	[173]
miR-122a	Cyclin G1	Tumour suppressor	[174]
miR-155	AT1R, TP53INP1	Oncogene	[175]
miR-210	HIF-1α	Oncogene	[176]
miR-221/222	CD117	Oncogene	[177]
miR-205	PRKCε	Tumour suppressor	[178]
miR-20a	TIMP2, ATG7	Oncogene	[179]
miR-29b	Mcl-1	Oncogene	[180]
miR-712	TIMP3	Oncogene	[181,182]
